# *De novo* genome assembly of the white-spotted flower chafer (*Protaetia brevitarsis*)

**DOI:** 10.1093/gigascience/giz019

**Published:** 2019-04-05

**Authors:** Kui Wang, Pengpeng Li, Yongyang Gao, Chunqin Liu, Qinglei Wang, Jiao Yin, Jie Zhang, Lili Geng, Changlong Shu

**Affiliations:** 1 State Key Laboratory for Biology of Plant Diseases and Insect Pests, Institute of Plant Protection, Chinese Academy of Agricultural Sciences, No. 2, West Yuan Ming Yuan Road, Haidian District, Beijing 100193, P. R. China; 2 Beijing Sinobiocore Biological Technology Co., Ltd., No. 99, Kechuang 14th Street, Daxing District, Beijing 1001111, P. R. China; 3 Cangzhou Academy of Agricultural and Forestry Sciences, No. 18, West Jiuhe Road, Yunhe District, Cangzhou 061001, P. R. China

**Keywords:** *Protaetia brevitarsis*, white-spotted flower chafer, genome, assembly

## Abstract

**Background:**

*Protaetia brevitarsis*, commonly known as the white-spotted flower chafer, is an important Scarabaeidae insect that is distributed in most Asian countries. Recently, research on the insect's harmfulness to crops, usefulness in agricultural waste utilization, edibility, medicinal value, and usability in insect immunology has provided sufficient impetus to demonstrate the need for a detailed study of its biology. Herein, we sequenced the whole genome of this species to improve our understanding and study of *P. brevitarsis*.

**Findings:**

We developed a highly reliable genome resource for *P. brevitarsis* (Lewis, 1879; Coleoptera: Cetoniinae) using Illumina and PacBio sequencing platforms. A total of 135.75 gigabases (Gb) was generated, providing 150-fold coverage based on the 810-megabases (Mb) estimated genome size. The assembled *P. brevitarsis* genome was 751 Mb (including the scaffolds longer than 2 kilobases (kb)) with 327 scaffolds, and the N50 length of the assembly was 2.94 Mb. A total of 34,110 (22,229 in scaffolds and 11,881 located in alleles) genes were identified using Evidence Modeler, which was based on the gene prediction results obtained from 3 different methods (*ab initio*, RNA sequencing based, and known gene based).

**Conclusions:**

We assembled a high-quality *P. brevitarsis* genome, which will not only provide insight into the biology of the species but also provide a wealth of information that will inform researchers on the evolution, control, and utilization of *P. brevitarsis*.

## Data Description

### Context

**Figure 1:**
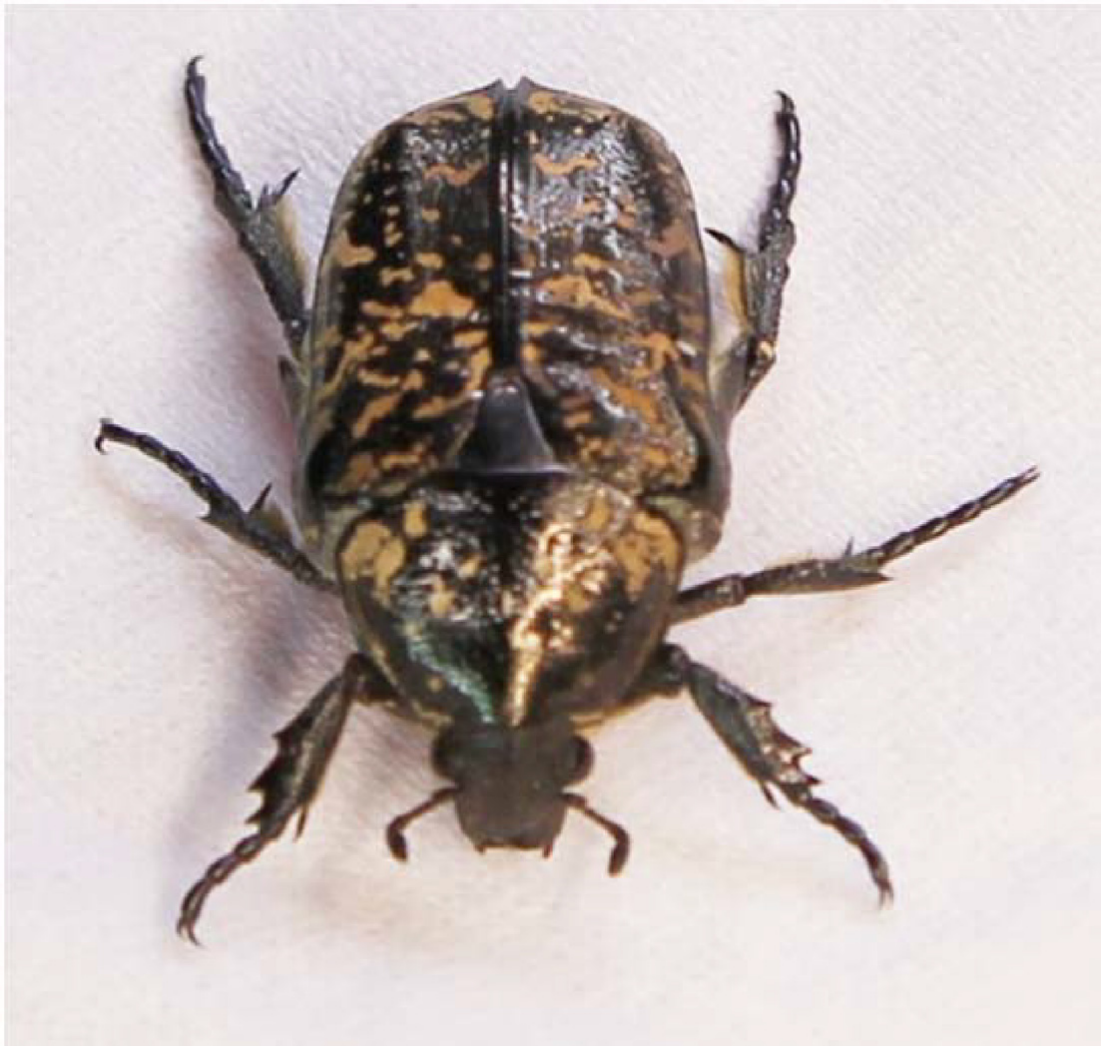
Image of adult of the white-spotted flower chafer, *P. brevitarsis*.

*Protaetia brevitarsis* (*Protaetia brevitarsis*, NCBI:txid348688), commonly known as the white-spotted flower chafer (Fig. 1), is an important Scarabaeidae insect that is distributed throughout China and surrounding countries (Mongolia, Russia, Japan, South Korea, and North Korea) [1]. *P. brevitarsis* adults feed on multiple plant parts, while larvae live in the topsoil and feed on soil humus, decaying plant residues, and even animal dung. *P. brevitarsis* adults represent one of the most destructive pests in agriculture, and these insects cause direct damage to ≥29 important plant species [2]. In contrast, *P. brevitarsis* larvae are considered resource insects, and researchers in China investigated the use of the larvae to convert crop straw and other agricultural wastes to organic fertilizer [3]. Furthermore, research examined the potential of the insects to mitigate pollution caused by the improper treatment of crop straw and to produce insect protein fodder. In South Korea, *P. brevitarsis* was recently registered as a temporal standard food ingredient by the Ministry of Food and Drug Safety, and the insects were mass reared for commercial purposes [4,5]. Larval stage insects have been used in traditional medicine to treat inflammatory disease, breast cancer, hepatic cancer, liver cirrhosis, and hepatitis. Furthermore, researchers have identified and characterized compounds that were associated with activity against microbial pathogens [6] and cancer cells [7, 8], as well as those that inhibited platelet aggregation or thrombosis [9]. Furthermore, *P. brevitarsis* larvae are also considered a good model for insect immune system studies [10–12]. *P. brevitarsis* have well-developed cellular and humoral defence systems, and *P. brevitarsis* last instar larvae can produce approximately 0.5 mL of haemolymph, which is sufficient for most immunological experiments.

These significant properties provided enough impetus for a detailed study of *P. brevitarsis* biology. However, the genetic basis and the evolutionary characteristics of *P. brevitarsis* remain unclear, and little information about this insect is available in public databases. In this study, we provide the first report of the draft *P. brevitarsis* genome assembly with high sequencing depth coverage that was generated using the Illumina and PacBio genome-sequencing platforms. These data will provide valuable information for further studies, as well as the control or utilization of this insect.

### Samples and sequencing

A single *P. brevitarsis* pupa was selected from the laboratory population for genome sequencing. The laboratory population was derived from a field population collected in Gongzhuling, Jilin province, China. The genomic DNA of the pupa was extracted using a Qiagen Blood and Tissue Kit (Qiagen, Valencia, CA, USA) according to the manufacturer's instructions. A 20-kb SMRTbell library was generated using a BluePippin DNA Size Selection instrument (Sage Science, Beverly, MA, USA), and the prepared library was sequenced using P6/C4 chemistry according to the manufacturer's protocols (Pacific Biosciences, Menlo Park, CA, USA). The single-molecule real-time (SMRT) sequencing of long reads was conducted on a PacBio RS II System, and we obtained 27.98 Gb PacBio data (Table 1).

**Table 1.** Summary statistics of generated sequence data

Library name	Experiment title	Sequencing instrument	Total bases (bp)	Accession No.
Raw_200_DNA_Hiseq	DNA pair end (PE) library	Illumina HiSeq 2500	48,637,157,380	-
Raw_420_DNA_Hiseq	DNA PE library	Illumina HiSeq 2500	59,133,181,272	-
Filtered_200_DNA_Hiseq	DNA PE library	Illumina HiSeq 2500	46,322,512,285	SRR7421508
Filtered_420_DNA_Hiseq	DNA PE library	Illumina HiSeq 2500	40,349,624,172	SRR7421507
DNA_PacBio1	DNA PacBio library	PacBio RS II	1,248,598,019	SRR7429397
DNA_PacBio2	DNA PacBio library	PacBio RS II	1,742,919,487	SRR7429396
DNA_PacBio3	DNA PacBio library	PacBio RS II	1,471,376,296	SRR7429395
DNA_PacBio4	DNA PacBio library	PacBio RS II	1,446,032,590	SRR7429394
DNA_PacBio5	DNA PacBio library	PacBio RS II	1,410,533,432	SRR7429401
DNA_PacBio6	DNA PacBio library	PacBio RS II	1,303,543,797	SRR7429400
DNA_PacBio7	DNA PacBio library	PacBio RS II	1,185,731,970	SRR7429399
DNA_PacBio8	DNA PacBio library	PacBio RS II	1,360,241,545	SRR7429398
DNA_PacBio9	DNA PacBio library	PacBio RS II	1,033,036,210	SRR7429403
DNA_PacBio10	DNA PacBio library	PacBio RS II	981,818,132	SRR7429402
DNA_PacBio11	DNA PacBio library	PacBio RS II	1,192,589,806	SRR7429389
DNA_PacBio12	DNA PacBio library	PacBio RS II	707,437,407	SRR7429388
DNA_PacBio13	DNA PacBio library	PacBio RS II	659,418,664	SRR7429391
DNA_PacBio14	DNA PacBio library	PacBio RS II	618,638,129	SRR7429390
DNA_PacBio15	DNA PacBio library	PacBio RS II	630,384,409	SRR7429393
DNA_PacBio16	DNA PacBio library	PacBio RS II	761,167,622	SRR7429392
DNA_PacBio17	DNA PacBio library	PacBio RS II	2,180,394,708	SRR7470031
DNA_PacBio18	DNA PacBio library	PacBio RS II	2,035,388,872	SRR7470028
DNA_PacBio19	DNA PacBio library	PacBio RS II	1,796,143,706	SRR7470027
DNA_PacBio20	DNA PacBio library	PacBio RS II	1,980,034,243	SRR7470030
DNA_PacBio21	DNA PacBio library	PacBio RS II	2,229,575,050	SRR7470029
Egg	RNA-Seq library	Illumina HiSeq 2500	6,049,557,600	SRR7418793
Larva	RNA-Seq library	Illumina HiSeq 2500	6,112,599,900	SRR7418797
Prepupal	RNA-Seq library	Illumina HiSeq 2500	6,168,021,600	SRR7418791
Middle pupal	RNA-Seq library	Illumina HiSeq 2500	6,015,743,700	SRR7418789
Late pupal	RNA-Seq library	Illumina HiSeq 2500	6.260,516,400	SRR7418796
Male adult	RNA-Seq library	Illumina HiSeq 2500	6.054,195,300	SRR7418798
Female adult	RNA-Seq library	Illumina HiSeq 2500	6.188,099,400	SRR7418790
Forewing (D1)	RNA-Seq library	Illumina HiSeq 2500	6.234,580,800	SRR7585362
Forewing (D3)	RNA-Seq library	Illumina HiSeq 2500	6.208,411,800	SRR7418792
Underwing (D1)	RNA-Seq library	Illumina HiSeq 2500	6.154,223,400	SRR7418801
Underwing (D3)	RNA-Seq library	Illumina HiSeq 2500	6.172,792,500	SRR7418794
Head (D1)	RNA-Seq library	Illumina HiSeq 2500	6.090,345,900	SRR7418799
Head (D3)	RNA-Seq library	Illumina HiSeq 2500	6.247,745,100	SRR7418800

Note: D1 or D3: tissues of newly (1-day) or 3-day emerged adults.

Furthermore, 2 paired-end libraries with insert sizes of 200 and 420 bp, respectively, were constructed using the TruSeq DNA PCR-Free Library Prep Kit, and sequencing was performed on an Illumina HiSeq 2500 sequencer (Illumina, San Diego, CA, USA), producing 107.77 Gb of raw data (Table 1). The following reads were then removed: (1) reads with Ns, >20% low-quality bases (quality criterion: Q20), or >10 bp that overlapped with adapter sequences (allowing ≤3 bp mismatches) and (2) duplicated reads generated by polymerase chain reaction amplification during library construction. Therefore, a total of 86.67 Gb of clean data were obtained (Table 1). For transcriptome sequencing, total RNA from *P. brevitarsis* whole eggs, larvae, 3 different pupal stages, male adults, female adults, and tissues (forewing, underwing, and head) of newly (1-day) and 3-day emerged adults were collected and prepared using TRIzol reagent (Invitrogen, CA, USA). RNA quality was confirmed by gel electrophoresis, and the quantity was determined using a Nanodrop spectrophotometer. Sequencing libraries were generated using an Illumina TruSeq Stranded mRNA Library Prep Kit (Illumina, CA, USA), and sequencing was also performed on an Illumina HiSeq 2500 sequencer. In total, 79.96 Gb of data (Table 1), comprising 533.05 million reads (Table 2), were generated.

**Table 2.** Summary statistics of RNA-Seq reads mapped onto the assemblies

Sample	No. of reads	Reads mapped to scaffolds (No. [%])	Reads mapped to ASs (No. [%])
Egg	40,330,384	35,659,462 (88.42)	14,961,772 (37.10)
Larva	40,750,666	33,467,876 (82.13)	15,266,678 (37.46)
Prepupal stage	41,120,144	34,780,542 (84.58)	15,172,926 (36.90)
Middle pupal stage	40,104,958	36,742,877 (91.62)	15,307,418 (38.17)
Late pupal stage	41,736,776	37,468,206 (89.77)	17,178,294 (41.16)
Male adult	40,361,302	36,198,806 (89.69)	14,518,842 (35.97)
Female adult	41,253,996	32,620,778 (79.07)	16,135,954 (39.11)
Forewing (D1)	41,563,872	36,449,354 (87.69)	9233,440 (22.22)
Forewing (D3)	41,389,412	35,727,909 (86.32)	13,516,032 (32.66)
Underwing (D1)	41,028,156	36,669,771 (89.38)	14,943,970 (36.42)
Underwing (D3)	41,151,950	37,484,048 (91.09)	16,851,062 (40.95)
Head (D1)	40,602,306	32,278,844 (79.50)	11,935,160 (29.40)
Head (D3)	41,651,634	35,214,660 (84.55)	11,779,198 (28.28)

Note: D1 or D3: tissues of newly (1-day) or 3-day emerged adults.

### Genome size and heterozygosity estimation

**Figure 2:**
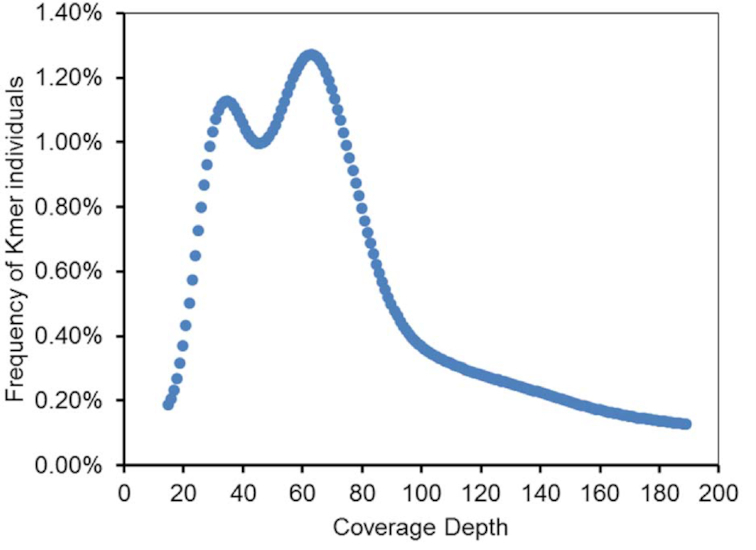
The 17-mer distribution of the *P. brevitarsis* genome using the jellyfish [13] program with 420-bp paired-end whole-genome sequencing data.

The k-mer analysis approach was used to estimate the genome size and heterozygosity. Quality-filtered 420 bp–insert size clean reads (Illumina) were used to perform the k-mer (k = 17) analysis. A total of 60,101,962,676 k-mers were counted from these clean reads. The count distribution of 17-mers with the highest peak occurred at a depth of 63 (Fig. 2), the estimated genome size was ∼810 Mb, and the heterozygosity was 2.35% (Table S1).

### Genome assembly

The k-mer analysis indicated that the *P. brevitarsis* genome exhibited high heterozygosity, and a hierarchical assembly stratagem was used for genome assembly. Allele sequences (ASs) that differentiated from different sister chromatids could potentially generate bubbles and junctions in the string graph, which would hinder the genome assembler's generation of longer contigs. To achieve a high-quality assembly, we used PacBio long reads during the assembly process, and we detected and separated ASs during the assembly process in the hierarchical stratagem.

Before assembly, all PacBio reads were quality filtered using SMRT Portal, and polymerase reads with read score <0.80 and subread lengths shorter than 500 bp were removed. After data filtering, 14.25 Gb of PacBio subreads were left (Table 3). The N50 value and mean size of filtered PacBio subreads were 16.06 and 10.53 kb, respectively, and the mean read score of filtered PacBio subreads was 0.837.

**Table 3.** Summary statistics of data during the assembly process

	No.	Total bases (bp)	N50	Mean length (bp)
Filtered PacBio reads	1,353,926	14,251,368,546	16,059	10,525
Elementary contigs	8,760	1,127,134,570	190,967	128,668
HGCs	3,816	738,878,186	347,620	193,626
ASs	4,939	391,445,919	91,687	79,256
Scaffolds	313	751,076,257	2,939,522	2,399,604
Corrected HGCs	3,821	739,117,100	327,214	193,435
Corrected ASs	4,939	393,190,609	92,105	79,609
Corrected scaffolds	313	751,076,257	2,939,522	2,399,604
Final scaffolds	327	750,736,501	2,939,521	2,295,830

The mitochondrial genome was assembled first. The mitochondrial genome reads were picked out by alignment to the published reference *P. brevitarsis* mitochondrial genome (Genebank: NC_02 3453.1) using Blasr (BLASR, RRID:SCR_000764) (Table S2). Then, the selected reads were assembled using Canu (Canu, RRID:SCR_015880) (Table S2). When comparing the new assembled mitochondrial genome with the previous one (Genebank: NC_02 3453.1), there were 116 single-nucleotide variations and 12 insertions or deletions.

**Figure 3:**
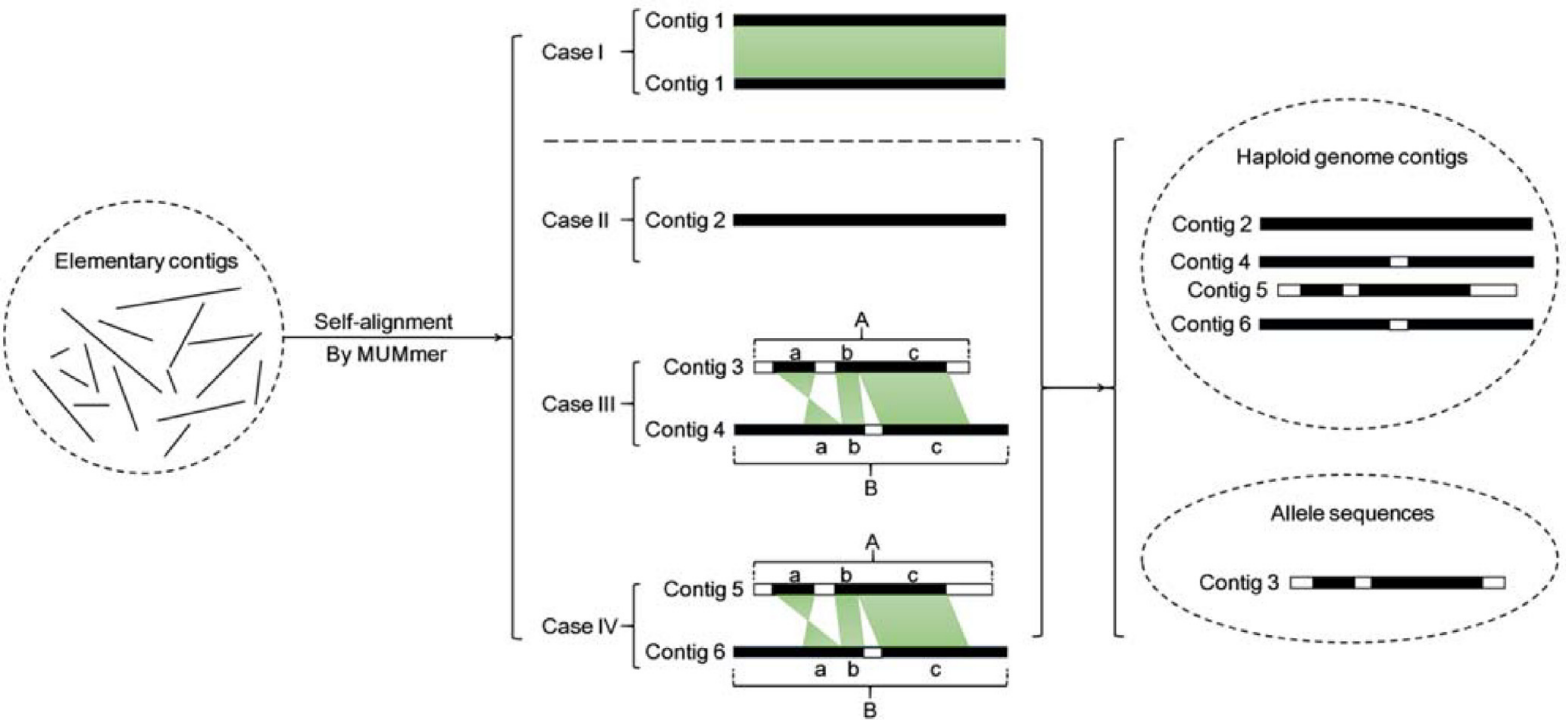
Schematic illustration of the method used to detect aligned sequences in the assembly. MUMmer was used to perform self-alignment on the elementary contigs; paired contigs were categorized into 4 types of outcome. In Case I, the contig aligns to itself, which will be ignored. In Case II, Contig 2 represents a contig with no obvious alignment with other contigs, and the contig type is defined as haploid genome contig. In Cases III and IV, the contig under analysis can align with another contig; in the figure, Contigs 4 and 6 are defined as haploid genome contigs because B is longer than A. In Case III, Contig 3 (the shorter contig) is defined as AS because the aligned sequence (a+b+c) accounted for >85% of the no-repeat sequence total length (A). In Case IV, Contig 5 (the shorter contig) is considered to be a duplication of sequence because the aligned sequence (a+b+c) accounted for <85% of the no-repeat sequence total length (A); therefore, Contig 5 is defined as a haploid genome contig.

Then, we used Marvel (Table S2) [14] to construct string graphs of filtered PacBio reads, and we assembled them into unitigs. In this step, both unitigs and singletons were collected as elementary contigs, and the total size of the elementary contigs was 1,127,134,570 bp (N50 = 190,967 bp; Table 3). We then selected ASs and employed a whole-genome alignment strategy to recognize alternative heterozygous ASs after masking all repeat sequences in the elementary contigs. As shown in Fig. 3, MUMmer (Table S2) [15] was used conduct whole-genome self-alignments. Small individual matches were clustered using the longest increasing subset algorithm and were then merged into larger matches. These matches were used to calculate the coverage of overlapping lengths of each pair of elementary contigs. The short one was defined as the AS if 85% no-repeat sequence of the total length was aligned to the long elementary contigs or if 85% of the reads were the same as longer elementary contigs, while the longer one was kept in elementary contigs. Each AS was confirmed via dot plot examination, and sequences were used to restore the AS to elementary contigs if the alignment quality was poor. After this step, elementary contigs were separated into 2 parts, haploid genome contigs (HGCs) and the ASs. Finally, 3,816 HGCs were retained (N50 = 347,620 bp; total length = 738,878,186 bp), and 4,939 ASs were retained (N50 = 91,687 bp; total length = 391,445,919 bp) (Table 3). HGCs were joined and elementary scaffolds were produced using SSPACE (SSPACE, RRID:SCR_005056) (Table S2) [16] and all PacBio RSII subread information. With the above procedure, we obtained a haploid genome assembly with a size of 751.08 MB, 313 raw scaffolds, and an N50 scaffold size of 2.94 Mb (Table 3). In the last step, we used Pilon (Pilon, RRID:SCR_014731) (Table S2) [17] to correct single-base differences, small insertions or deletions, block substitution events, and gaps in HGCs, ASs, and elementary scaffolds. All Illumina genome sequence data were aligned using BWA (BWA, RRID:SCR_010910) (Table S2) [18], and the corresponding alignments were provided as input to Pilon to conduct consensus polishing. Finally, the total size of the corrected HCGs and ASs was 739.12 Mb (including 3,821 contigs) and 393.19 Mb (including 4,939 sequences), respectively. The total size of the corrected scaffolds was 751.08 Mb (including 313 scaffolds), and the N50 was 2.94 Mb (Table 3). Then, we ran the assembly sequences through Contamination Screen and removed the contaminated sequences, trimming any Ns at the ends of the sequence. The total size of the final scaffolds was 750.74 Mb (including 327 scaffolds), and the N50 was 2.94 Mb (Table 3).

### Validation and quality control

The completeness and accuracy of the assembly were assessed using 3 independent measures. We first mapped all Illumina paired-end reads onto the assemblies (scaffolds and ASs), and the results indicated that >73.24-fold effective depth was obtained across all of the scaffolds. Regarding ASs, the lowest depth was 13.08-fold. These data indicated that the genome was extensively covered by sequence reads (Table 4). We then aligned RNA-sequencing (RNA-Seq) reads to our assemblies (scaffold and ASs) using Spliced Transcripts Alignment to a Reference (STAR) (STAR, RRID:SCR_015899) (Table S2) [19]. For the RNA-Seq reads, the data indicated that 79.07–91.62% of reads generated from these samples could be correctly mapped to the scaffolds with appropriate splicing, while 22.22–41.16% of RNA-Seq reads were mapped to the ASs (Table 2). Furthermore, the benchmarking universal single-copy orthologs (BUSCO, RRID:SCR_015008) (BUSCO, Table S2) [20] data set was used to evaluate the completeness of the assembly. Approximately 93% of complete BUSCOs were found in the assembly. When compared to other sequenced coleopteran genomes, the data indicated that the complete BUSCOs found in the current assembled *P. brevitarsis* genome totaled 93.00%. Therefore, this percentage was lower than that observed in *Tribolium castaneum* (96.59%) and *Pyrocoelia pectoral* (98.80%) but higher than that observed in other genomes (Table 5). In summary, these results suggested that the genome assembly was complete and of high quality.

**Table 4.** Summary statistics of Illumina genome-sequencing reads mapped onto the assemblies

	Mean depth	Lowest depth	Highest depth
Corrected HGCs	121.9	73.24	167.07
Corrected ASs	85.63	13.08	1,221.48
Corrected scaffolds	122.2	73.24	167.07

**Table 5.** BUSCOs found in coleopteran genomes

Species	Complete (%)	Fragment (%)	Missing (%)	Duplication (%)
*O. taurus*	80.45	10.00	9.55	8.80
*D. ponderosae*	81.47	8.53	10.00	10.87
*A. glabripennis*	82.31	8.70	8.99	9.12
*A. planipennis*	91.90	2.70	5.40	4.10
*P. brevitarsis*	93.00	1.90	5.10	7.20
*T. castaneum*	96.59	2.90	0.51	9.40
*P. pectoral*	98.80	0.60	0.60	7.20

### Genome annotation

Repetitive sequences, including tandem repeats and interspersed repeats, were searched for in the *P. brevitarsis* genome. Tandem repeats in the genome were defined as ≥2 adjacent, approximate copies of a pattern of nucleotides. Tandem Repeats Finder (Table S2) [21] was used to search for tandem repeats in the genome. Two independent methods, homology based and *de novo* prediction, were used to identify interspersed repeats in the assembly. Regarding the homology-based method, the assembled genome was compared with Repbase (V.22.11) [22] using RepeatMasker (RepeatMasker, RRID:SCR_012954) and RepeatProteinMasker (Table S2) with default settings [23]. For *de novo* predictions, we built a *de novo* repeat library with LTR Finder (Table S2) [24] and RepeatScout (RepeatScout, RRID:SCR_014653) (Table S2) [25]. RepeatProteinMask (Table S2) was then used to identify putative transposable element (TE)-related proteins. After merging all of the repetitive elements identified using the aforementioned tools, we identified a total of 396.23 Mb of repetitive sequences, accounting for 51.82% of the haploid genome (Table 6). Regarding the ASs, 220.22 Mb of repetitive sequences were identified, accounting for 56.02% of the total length of the genome (Table 6).

**Table 6.** Summary of identified repeat elements in the *P. brevitarsis* genome

Repeat element	Repeat elements from haploid genome		Repeat elements from ASs	
	Length (bp)	Percentage (%)	Length (bp)	Percentage (%)
Long terminal repeat	109,722,085	14.35	60,133,491	15.29
Long interspersed nuclear element	101,529,627	13.28	52,758,849	13.42
Short interspersed nuclear element	259,936	0.03	50,366	0.01
DNA element	166,788,392	21.81	92,972,801	23.65
Simple repeat	4,749,908	0.62	2,485,661	0.63
Low complexity	1,132,919	0.15	656,626	0.17
Rolling circle	7,162,276	0.94	5,220,692	1.33
Satellite	304,734	0.04	221,437	0.06
Other	131,605	0.02	99,113	0.03
Unclassified	4,451,277	0.58	5,618,712	1.43
Total	396,232,759	51.82	220,217,748	56.02

Four types of noncoding RNAs were searched for across the *P. brevitarsis* genome. Transfer RNAs (tRNAs) were annotated using tRNAscan-SE (tRNAscan-SE, RRID:SCR_010835) (Table S2) [26] with default parameters for eukaryotes. Ribosomal RNAs (rRNAs) were identified using BlastN (BLASTN, RRID:SCR_001598) alignments, and RNAmmer (Table S2) [27] was used to predict rRNAs and their subunits. Small nuclear RNAs and microRNAs were predicted using the Rfam (Rfam, RRID:SCR_007891) [28] database and BlastN (Table S2). These analyses identified 864 microRNAs, 3,277 tRNAs, 113 rRNAs, and 95 small nuclear RNAs.

The protein-coding genes were annotated on the basis of evidence obtained using the homology-based method, *ab initio* prediction, and RNA-Seq data. Regarding the homology-based method, protein sequences from all Coleoptera in the NCBI Reference Sequence Database (2 October 2017) were collected and aligned with our genome scaffolds using GenBlastA (Table S2) [29]. Target regions were then expanded to 10 kb both for upstream and downstream analyses and were then used to determine accurate gene structures using GeneWise (GeneWise, RRID:SCR_015054) software (Table S2) [30]. For *de novo* prediction, AUGUSTUS (Augustus, RRID:SCR_008417) (Table S2) [31], Genemark (GeneMark, RRID:SCR_011930) (Table S2) [32], and SNAP (SNAP, RRID:SCR_007936) (Table S2) [33] programs were used to obtain predicted gene structures from repeat-masked genomes. The top 300 longest coding sequence identities (>90%) associated with RNA-Seq unigenes were selected to train these programs, and the resulting suitable parameters were used for *P. brevitarsis* gene *de novo* prediction. Furthermore, we identified gene structures with the assistance of RNA-Seq data. First, RNA-Seq reads were aligned against the genome using STAR (Table S2) to identify candidate exon regions with default parameters. StringTie (StringTie, RRID:SCR_016323) (Table S2) [34] was then used to assemble the aligned reads into transcripts. Finally, all data were combined using Evidence Modeler (EVidenceModeler, RRID:SCR_014659) [35] to produce the consensus gene set, and 22,229 and 11,881 protein-coding genes were generated from scaffolds and ASs, respectively. There were 469 identical genes detected between the 2 methods.

Functional annotation of genes was performed using BlastP (BLASTP, RRID:SCR_001010) (Table S2) alignment to the Kyoto Encyclopedia of Genes and Genomes (KEGG) (KEGG, RRID:SCR_012773) [36, 37], Nr/Nt (2 March 2016), [38], Swiss-Prot/Uniprot and TrEMBL databases [39, 40]. Motifs and domains were determined using InterProScan (InterProScan, RRID:SCR_005829) [41, 42] against protein databases, including Pfam (Pfam, RRID:SCR_004726) [43, 44], SMART (SMART, RRID:SCR_005026) [45, 46], PANTHER (PANTHER, RRID:SCR_004869) [47, 48], and PROSITE (PROSITE, RRID:SCR_003457) [49, 50]. The results indicated that 17,625 genes from the haploid genome were annotated, while 8,887 genes from ASs were annotated (Table 7).

**Table 7.** Summary of annotated genes in the *P. brevitarsis* genome

Database	Annotated genes	
	From haploid genome (No. [%])	From ASs (No. [%])
KEGG	15,828 (71.16)	7,980 (67.17)
Swiss-Prot	10,509 (47.25)	5,179 (43.59)
Nr	17,487 (78.62)	8,757 (73.71)
Nt	3,688 (16.58)	1,855 (15.61)
TrEMBL_eggNOG	15,986 (71.87)	8,029 (67.58)
No. of total annotated genes	17,625 (79.24)	8,887 (74.80)

### Phylogenetic tree reconstruction and divergence time estimation

**Figure 4:**
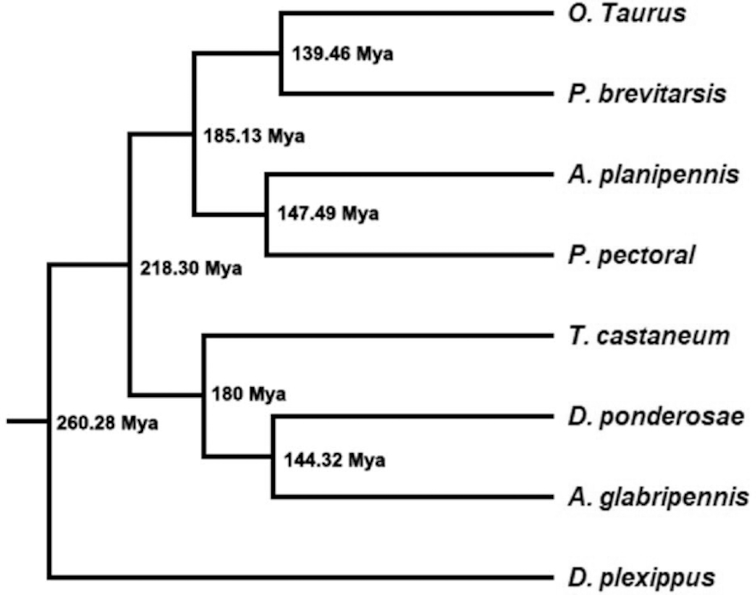
Phylogenetic relationship of *P. brevitarsis* and 6 Coleoptera insects based on 2,354 orthologue genes. Estimated divergence times using *D. ponderosae-T. castaneum* [180Mya] as the calibration time are shown [55].

To investigate the phylogenetic position of *P. brevitarsis*, protein data from the NCBI database were retrieved for coleopteran insects *Anoplophora glabripennis, Dendroctonus ponderosae, T. castaneum, Onthophagus taurus, P. pectoral*, and *Agrilus planipennis*, and the lepidopteran insect *Danaus plexippus* was used to root the tree. All proteins were pooled together, and OrthoMCL (Table S2) [51] was used for orthologue group identification. A total of 76,623 orthologue groups were identified, and 13,627 gene families were specific to *P. brevitarsis*. Moreover, 2,354 orthologue groups, which were identified as single-copy genes that were shared between these species, were selected for subsequence analyses. The selected proteins from these species were concatenated and subjected to multiple alignment using MAFFT (MAFFT, RRID:SCR_011811) (Table S2) [52] and profile trimming with TrimAI (Table S2) [53]. After that, Beast 2 (Table S2) [54] was used to conduct phylogenetic analyses. The phylogenetic tree indicated that *P. brevitarsis* was closely related to *O. taurus*, and the estimated divergence time was ∼140 million years ago (Fig. 4).

## Discussion

Scarabaeoidea is a diverse lineage of predominantly plant- and dung-feeding beetles that consists of >31,000 described species [55]. In this study, we sequenced the genome of *P. brevitarsis*, and this represents the first high-quality genome of a plant-feeding scarab. Plant- and dung-feeding scarab beetles are considered sister lineages [56], and they exhibit modes that can be used to test hypotheses of species diversification that may have been driven by interactions with angiosperm and mammal lineages. Therefore, *P. brevitarsis* genomic data could provide useful resources for studies that examine the evolution of insect lineages and major biotic changes in Earth's history. Furthermore, this high-quality reference genome will contribute to research associated with several recent investigations regarding *P. brevitarsis*’ harmfulness to crops, usefulness in agricultural waste utilization, edibility, medicinal value, and applications to insect immunology research.

## Availability of supporting data

Raw sequencing reads have been deposited in the Sequence Read Archive database with NCBI Bioproject ID PRJNA477715 and PRJNA482477. The genome assembly including haploid genome contigs, ASs, and complete mitochondrial genome has been deposited in NCBI Genomes with accession No. RXPK00000000. Gene models and other supporting data are available via the *GigaScience* database GigaDB [57]. Key parameters we used that may affect the software results are available in Table S2.

## Additional files

Table S1. Estimation of genome characteristics based on 17-mer analysis.

Table S2. The software used in the study.

## Abbreviations

AS: allele sequence; bp: base pair; BUSCO: benchmarking universal single-copy ortholog; Gb: gigabases; HGC: haploid genome contig; kb: kilobases; KEGG: Kyoto Encyclopedia of Genes and Genomes; Mb: megabases; NCBI: National Center for Biotechnology Information; RNA-Seq: RNA sequencing; SMRT: single-molecule real time; STAR: Spliced Transcripts Alignment to a Reference; rRNA: ribosomal RNA; TE: transposable element; tRNA: transfer RNA.

## Competing interests

The authors declare that they have no competing interests.

## Funding

This study was supported by the National Key Research and Development Program of China (2018YFD0800906 and 2017YFD0201204) and National Natural Science Foundation of China (31530095 and 31872935).

## Author contributions

C.S. and J.Z. designed the study; C.L. and Q.W. collected samples; C.L., J.Y., and L.G. extracted DNA and RNA samples; Y.G. and P.L. worked on sequencing; C.S., P.L., and K.W. worked on the genome assembly, assessment, and annotation; and C.S. and K.W. wrote the manuscript. All authors read and approved the final version of the manuscript.

## Supplementary Material

GIGA-D-18-00277_Original_Submission.pdf

GIGA-D-18-00277_Revision_1.pdf

Response_to_Reviewer_Comments_Original_Submission.pdf

Reviewer_1_Report_Original_Submission -- Eduardo Enrique Zattara, Ph.D. 8/26/2018 Reviewed

Reviewer_2_Report_Original_Submission -- Bent Petersen 11/26/2018 Reviewed

Reviewer_2_Report_Revision_1 -- Bent Petersen 1/16/2019 Reviewed

Supplemental File
