# Supplementary material for: De novo genome assembly of the white-spotted flower chafer (Protaetia brevitarsis)
Source: Gigascience. 2019 Apr 5;8(4):giz019. doi: 10.1093/gigascience/giz019 (PMC6449472; doi:10.1093/gigascience/giz019)
Supplement: GIGA-D-18-00277_Original_Submission.pdf [file giz019_giga-d-18-00277_original_submission.pdf]

GigaScience

De novo genome assembly of the white-spotted flower chafer (Protaetia brevitarsis)

--Manuscript Draft--

**Manuscript Number:**	GIGA-D-18-00277
**Full Title:**	De novo genome assembly of the white-spotted flower chafer (Protaetia brevitarsis)
**Article Type:**	Data Note
**Funding Information:**	National Key Research and Development Program of China (2017YFD0201204) — Not applicable<br>National Key Research and Development Program of China (2017YFD0200604) — Not applicable<br>National Natural Science Foundation of China (Nos. 31530095) — Not applicable<br>National Natural Science Foundation of China (Nos. 41701298) — Not applicable
**Abstract:**	Background: Protaetia brevitarsis, commonly known as the white-spotted flower chafer, is an important Scarabaeidae insect that is distributed in most Asian countries. Recently, research on the insect’s harmfulness on crops, usefulness in agricultural waste utilization, edibleness, medicinal value, and usability in insect immunology has provided sufficient impetus for a detailed study of its biology. Here, we sequenced the whole genome of this species to improve our understanding and study of P. brevitarsis. Findings: We developed a highly reliable genome resource for P. brevitarsis (Lewis, 1879; Coleoptera: Cetoniinae) using Illumina and PacBio sequencing platforms. A total of 135.75 gigabases (Gb) was generated, providing 150-fold coverage based on the 810-megabases (Mb) estimated genome size. The assembled P. brevitarsis genome was 751 Mb (>2 kilobases (kb)) with 313 scaffolds, and the N50 length of the assembly was 2.94 Mb. A total of 34,473 genes were identified using Evidence Modeler, which was based on the gene prediction results obtained from three different methods (ab initio, RNA-seq-based, and known-gene-based). Conclusions: We assembled a high-quality P. brevitarsis genome, which will not only provide insight into the biology of the species, but it will also provide a wealth of information that will inform researchers on the evolution, control, and utilization of P. brevitarsis.
**Corresponding Author:**	Changlong Shu, Ph.D.<br><br>Beijing, CHINA
**Corresponding Author Secondary Information:**	
**Corresponding Author's Institution:**	
**Corresponding Author's Secondary Institution:**	
**First Author:**	Kui Wang
**First Author Secondary Information:**	
**Order of Authors:**	Kui Wang<br>Pengpeng Li<br>Yongyang Gao<br>Chunqin Liu<br>Qinglei Wang<br>Jiao Yin<br>Jie Zhang

Powered by Editorial Manager® and ProduXion Manager® from Aries Systems Corporation

	Lili Geng
	Changlong Shu, Ph.D.
**Order of Authors Secondary Information:**	
**Additional Information:**	
**Question**	**Response**
Are you submitting this manuscript to a special series or article collection?	No
**Experimental design and statistics**<br><br>Full details of the experimental design and statistical methods used should be given in the Methods section, as detailed in our Minimum Standards Reporting Checklist. Information essential to interpreting the data presented should be made available in the figure legends.<br><br>Have you included all the information requested in your manuscript?	Yes
**Resources**<br><br>A description of all resources used, including antibodies, cell lines, animals and software tools, with enough information to allow them to be uniquely identified, should be included in the Methods section. Authors are strongly encouraged to cite Research Resource Identifiers (RRIDs) for antibodies, model organisms and tools, where possible.<br><br>Have you included the information requested as detailed in our Minimum Standards Reporting Checklist?	Yes
**Availability of data and materials**<br><br>All datasets and code on which the conclusions of the paper rely must be either included in your submission or deposited in publicly available repositories (where available and ethically appropriate), referencing such data using	Yes

Powered by Editorial Manager® and ProduXion Manager® from Aries Systems Corporation

a unique identifier in the references and in the “Availability of Data and Materials” section of your manuscript.<br><br>Have you have met the above requirement as detailed in our Minimum Standards Reporting Checklist?	

manuscript Click here to download Manuscript manuscript.doc

***De novo* genome assembly of the white-spotted flower chafer (*Protaetia brevitarsis*)**

Kui Wang[1†], Pengpeng Li[2†], Yongyang Gao[2], Chunqin Liu[3], Qinglei Wang[3], Jiao Yin[1], Jie Zhang[1], Lili Geng[1], and Changlong Shu[1*]

[1] State Key Laboratory for Biology of Plant Diseases and Insect Pests, Institute of Plant Protection, Chinese Academy of Agricultural Sciences, Beijing 100193, P. R. China

[2] Beijing Sinobiocore Biological Technology Co., Ltd., Beijing 100193, P. R. China

[3] Cangzhou Academy of Agricultural and Forestry Sciences, Cangzhou 061001, P. R. China

[*]Address correspondence to Changlong Shu (E-mail: clshu@ippcaas.cn, Tel: +86 10 62812642)

[†]These authors equally contributed and should be regarded as co-first authors.

Abstract

Background: *Protaetia brevitarsis*, commonly known as the white-spotted flower chafer, is an important Scarabaeidae insect that is distributed in most Asian countries. Recently, research on the insect's harmfulness on crops, usefulness in agricultural waste utilization, edibleness, medicinal value, and usability in insect immunology has provided sufficient impetus for a detailed study of its biology. Here, we sequenced the whole genome of this species to improve our understanding and study of *P. brevitarsis*.

Findings: We developed a highly reliable genome resource for *P. brevitarsis* (Lewis, 1879; Coleoptera: Cetoniinae) using Illumina and PacBio sequencing platforms. A total of 135.75 gigabases (Gb) was generated, providing 150-fold coverage based on the 810-megabases (Mb) estimated genome size. The assembled *P. brevitarsis* genome was 751 Mb (>2 kilobases (kb)) with 313 scaffolds, and the N50 length of the assembly was 2.94 Mb. A total of 34,473 genes were identified using Evidence Modeler, which was based on the gene prediction results obtained from three different methods (*ab initio*, RNA-seq-based, and known-gene-based). **Conclusions:** We assembled a high-quality *P. brevitarsis* genome, which will not only provide insight into the biology of the species, but it will also provide a wealth of information that will inform researchers on the evolution, control, and utilization of *P. brevitarsis*.

Keywords: *Protaetia brevitarsis*; white-spotted flower chafer; genome; assembly

Data Description

Context

Protaetia brevitarsis, commonly known as the white-spotted flower chafer (**Fig. 1**), is an important Scarabaeidae insect that is distributed throughout China and surrounding countries (Mongolia, Russia, Japan, South Korea, and north Korea) [1]. *P. brevitarsis* adults feed on multiple plants parts, while larvae live in the topsoil and feed on soil humus, decaying plant residues, and even animal dung. *P. brevitarsis* adults represent one of the most destructive pests in agriculture, and these insects cause direct damage to at least 29 important plant species [2]. In contrast, *P. brevitarsis* larvae are considered resource insects, and researchers in China investigated the use of the insects to convert crop straw and other agricultural wastes to organic fertilizer [3]. Furthermore, research examined the potential of the insects to mitigate pollution caused by the improper treatment of crop straw and to produce insect protein fodder. In Korea, *P. brevitarsis* was recently listed as a temporal food ingredient by the Korean Ministry of Food and Drug Safety, and the insects were mass reared for commercial purposes [4,5]. Larval stage insects have been used in traditional medicine to treat inflammatory disease, breast cancer, hepatic cancer, liver cirrhosis, and hepatitis. Furthermore, researchers identified and characterized effective compounds that were associated with activity against microbial pathogens [6] and cancer cells [7,8] as well as those that inhibited platelet aggregation or thrombosis [9]. Furthermore, *P. brevitarsis* larvae are also considered a good model for insect immune system studies [10–12]. *P. brevitarsis* have well-developed cellular and

humoral defence systems, and *P. brevitarsis* last instar larvae can produce approximately 0.5 mL of haemolymph, which is sufficient for most immunological experiments.

These significant properties provided enough impetus for a detailed study of *P. brevitarsis* biology. However, the genetic basis and the evolutionary characteristics of *P. brevitarsis* remain unclear, and very little information about this insect is available in public databases. In this study, we provide the first report of the draft *P. brevitarsis* genome assembly with high sequencing depth coverage that is generated using Illumina and PacBio genome sequencing platforms. These data will provide valuable information for further studies as well as the control or utilization of this insect.

Samples and sequencing

A single *P. brevitarsis* pupa was selected from the laboratory population for genome sequencing. The laboratory population was derived from a field population collected in Gongzhuling, Jilin province, China. The genomic DNA of the pupa was extracted using a Qiagen Blood and Tissue Kit (Qiagen, Valencia, CA, USA) according to the manufacturer’s instructions. A 20-kb SMRTbell library was generated using a BluePippin DNA Size Selection instrument (Sage Science, MA, USA), and the prepared library was sequenced using P6/C4 chemistry according to the manufacturer’s protocols (Pacific Biosciences, CA, USA). The single-molecule real-time sequencing of long reads was conducted on a PacBio RS II System, and we obtained 27.98 Gb PacBio data (**Table 1**).

Furthermore, two paired-end libraries with insert sizes of 200 bp and 420 bp,

respectively, were constructed using the TruSeq DNA PCR-Free Library Prep Kit, and sequencing was performed on an Illumina HiSeq 2500 sequencer (Illumina, San Diego, CA, USA), producing 107.77 Gb of raw data (**Table 1**). The following reads were then removed: (1) Reads with Ns, more than 20% low-quality bases, or more than 10 bp that overlapped with adapter sequences (allowing no more than 3 bp mismatches); and (2) Duplicated reads generated by PCR amplification during library construction. Therefore, a total of 86.67 Gb of clean data were obtained (**Table 1**). For transcriptome sequencing, total RNA from *P. brevitarsis* whole eggs, larvae, three different pupal stages, male adults, female adults, and tissues (forewing, underwing, and head) of newly (one-day) and three-day emerged adults were collected and prepared using TRIzol reagent (Invitrogen, CA, USA). RNA quality was confirmed by gel electrophoresis, and the quantity was determined using a Nanodrop spectrophotometer. Sequencing libraries were generated using an Illumina TruSeq Stranded mRNA Library Prep Kit (Illumina, CA, USA), and sequencing was also performed on an Illumina HiSeq 2500 sequencer. In total, 79.96 Gb of data (**Table1**), comprised of 533.05 million reads (**Table4**), were generated.

Genome size and heterozygosity estimation

The K-mer analysis approach was employed to estimate the genome size. Quality-filtered 420 bp-insert size clean reads (Illumina) were utilized to perform the K-mer (k = 17) analysis. A total of 60,101,962,676 K-mers were counted from these clean reads. The count distribution of 17-mers with the highest peak occurred at a depth of 63 (**Fig. 2**), and the estimated genome size was approximately 810 Mb

(**Table S1**).

Genome assembly

The *K-mer* analysis indicated that the *P. brevitarsis* genome exhibited high heterozygosity, and a hierarchical assembly stratagem was used for genome assembly. Allele sequences that differentiated from different sister chromatids could potentially generate bubbles and junctions in the string graph, which would hinder the genome assembler to generate longer contigs. To achieve a high-quality assembly, we used PacBio long reads during the assembly process, and we detected and separated allele sequences during the assembly process in the hierarchical stratagem.

Before assembly, all PacBio reads were quality-filtered using SMRT Portal v2.3.0, and polymerase reads with quality values lower than 0.80 and sub-read lengths shorter than 500 bp were removed. After data filtering, 14.25 Gb of PacBio sub-reads were left (**Table 2**). The N50 value and mean size of filtered PacBio sub-reads were 16.06 kb and 10.53 kb, respectively, and the average quality was 0.837. We then used Marvel (ba5a9d4) [13] with default parameters to construct string graphs of filtered PacBio reads, and we assembled them into unitigs. In this step, both unitigs and singletons were collected as elementary contigs, and the total size of the elementary contigs was 1,127,134,570 bp (N50 = 190,967 bp; **Table 2**). We then selected allele sequences and employed a whole-genome alignment strategy to recognize alternative heterozygous allele sequences after masking all repeat sequences in the elementary contigs. MUMmer v3.23 [14] (-mumreference -b 500 -g 200 -l 100) and Blast [15] were used conduct whole-genome self-alignments. Small individual matches were

clustered using the longest increasing subset algorithm (LIS), and were then merged into larger matches. These matches were used to calculate the coverage of overlapping lengths of each pair of elementary contigs. The short one if 85% no-repeat sequence of the total length was aligned to the long elementary contigs or if 85% of the reads was same as longer elementary contigs were defined as allele sequence (AS), while the longer one was kept in elementary contigs. Each allele sequence was confirmed via dot plot examination, and sequences were used to restore the allele sequence to elementary contigs if the alignment quality was poor. After this step, elementary contigs were separated into two parts, haploid genome contigs (HGCs) and the ASs. Finally, 3,816 HGCs were retained (N50 = 347,620 bp; total length = 738,878,186 bp), and 4,939 ASs were retained (N50 = 91,687 bp; total length = 391,445,919 bp) (**Table 2**). HGCs were joined and produced elementary scaffolds using SSPACE [16] and all PacBio RSII sub-read information. With the above procedure, we obtained a haploid genome assembly with a size of 751.08 MB, 313 raw scaffolds, and an N50 scaffold size of 2.94 Mb (**Table 2**). In the last step, we used Pilon [17] to correct single base differences, small indels, block substitution events and gaps in HGCs, ASs, and elementary scaffolds. All Illumina genome sequence data were aligned using BWA [18], and the corresponding alignments were provided as input to Pilon to conduct consensus polishing. Finally, the total size of the corrected HCGs and ASs was 739.12 Mb (including 3,821 contigs) and 393.19 Mb (including 4,939 sequences), respectively. And the total size of the corrected scaffolds was 751.08 Mb (including 313 scaffolds), and the N50 was 2.94 Mb (**Table 2**).

Validation and quality control

The completeness and accuracy of the assembly were assessed using three independent measures. We first mapped all Illumina paired-end reads onto the assemblies (scaffolds and allele sequences), and the results indicated that greater than 73.24-fold effective depth was obtained across all of the scaffolds. Regarding allele sequences, the lowest depth was 13.08-fold. These data indicated that the genome was extensively covered by sequence reads (**Table 3**). We then aligned RNA-seq reads to our assemblies (scaffold and allele sequences) using Spliced Transcripts Alignment to a Reference (STAR) with default parameters [19]. For the RNA-seq reads, the data indicated that all of RNA-seq reads generated from these samples could be correctly mapped to the scaffolds with appropriate splicing, while 22.22–41.16% of RNA-seq reads were mapped to the allele sequences (**Table 4**). Furthermore, the Benchmarking Universal Single-copy Orthologs (BUSCO, v1.1b1) [20] dataset was used to evaluate the completeness of the assembly. Approximately 93% of complete BUSCOs were found in the assembly. When compared to other sequenced coleopteran genomes, the data indicated that the complete BUSCOs found in the current assembled *P.brevitarsis* genome was 93%. Therefore, this percentage was lower than that observed in *Tribolium castaneum* (96.59%) and *Pyrocoelia pectoral* (98.80%), but it was higher than that observed in other genomes (**Table 5**). In summary, these results suggested that the genome assembly was complete and of high-quality.

Genome annotation

Repetitive sequences, including tandem repeats and interspersed repeats, were searched for in the *P. brevitarsis* genome. Tandem repeats in the genome were defined as two or more adjacent, approximate copies of a pattern of nucleotides. Tandem Repeats Finder (v. 4.07b) [21] was used to search for tandem repeats in the genome. Two independent methods, homology-based and *de novo* prediction, were used to identify interspersed repeats in the assembly. Regarding the homology-based method, the assembled genome was compared with Repbase (V.22.11) [22] using RepeatMasker (RepeatMasker, RRID:SCR 012954) and RepeatProteinMasker (v. 1.36) with default settings [23]. For *de novo* predictions, we built a *de novo* repeat library with long terminal repeats (LTR) Finder v. 1.0.5 (LTR Finder, RRID:SCR 015247) [24], and RepeatScout v. 1.0.5 (RepeatScout, RRID:SCR 014653) [25]. RepeatProteinMask was then used to identify putative transposable element (TE)-related proteins. After merging all of the repetitive elements identified using the aforementioned tools, we identified a total of 396.23 Mb of repetitive sequences, accounting for 51.82 % of the haploid genome (**Table 6**). Regarding the allele sequences, 220.22 Mb of repetitive sequences were identified, accounting for 56.02% of the total length of the genome (**Table 6**).

Four types of non-coding RNAs were searched for across the *P. brevitarsis* genome. Transfer RNAs (tRNAs) were annotated using tRNAscan-SE v1.3.1 (tRNAscan-SE, RRID: SCR 010835) [26] with default parameters for eukaryotes. Ribosomal RNAs (rRNAs) were identified using BlastN alignments, and RNAmmer v1.2 [27] was used to predict rRNAs and their subunits. Small nuclear RNAs

(snRNAs) and microRNAs (miRNAs) were predicted using the *Rfam* [28] database and BlastN (E-value ≤ 1e-5). These analyses identified 864 miRNAs, 3277 tRNAs, 113 rRNAs, and 95 snRNAs.

The protein-coding genes were annotated based on evidence obtained using the homology-base method, *ab initio* prediction, and RNA-seq data. Regarding the homology-based method, protein sequences from all Coleoptera in the NCBI Reference Sequence Database (2017-10-02) were collected and aligned with our genome scaffolds using GenBlastA [29] (E-value < 1e-5). Target regions were then expanded to 10 kb both for upstream and downstream analyses, and were then used to determine accurate gene structures using GeneWise software [30]. For *de novo* prediction, AUGUSTUS-3.2.2 [31], Genemark [32], and SNAP [33] programs were employed to obtain predicted gene structures from repeat-masked genomes. The top 300 longest CDS identities (higher than 90%) associated with RNA-Seq unigenes were selected to train these programs, and the resulting suitable parameters were used for *P. brevitarsis* gene *de novo* prediction. Furthermore, we identified gene structures with the assistance of RNA-seq data. Firstly, RNA-seq reads were aligned against the genome using STAR to identify candidate exon regions with default parameters. StringTie [34] was then utilized to assemble the aligned reads into transcripts. Finally, all data were combined using Evidence Modeler [35] to produce the consensus gene set, and 22,242 and 11,881 genes were generated from contigs and allele sequences, respectively. There were 469 identical genes detected between the two methods.

Functional annotation of genes was performed using BLASTP alignment to KEGG (http://www.genome.jp/kegg/) [36], Nr/Nt (2016-03-02, https://www.ncbi.nlm.nih.gov/), Swiss-Prot (http://us.expasy.org/sprot/) [37], and TrEMBL (https://www.ebi.ac.uk/uniprot/) [37] databases. Motifs and domains were determined using InterProScan (http://www.ebi.ac.uk/interpro/) [38] against protein databases, including Pfam (http://pfam.xfam.org/) [39], SMART (http://smart.embl-heidelberg.de/) [40], PANTHER (http://www.pantherdb.org/) [41], and PROSITE (http://www.expasy.ch/prosite/) [42]. The results indicated that 17,625 genes from the haploid genome were annotated, while 8,887 genes from allele sequences were annotated (**Table 7**).

Phylogenetic tree reconstruction and divergence time estimation

To investigate the phylogenetic position of *P. brevitarsis*, protein data from the NCBI database were retrieved for coleopteran insects *Anoplophora glabripennis*, *Dendroctonus ponderosae*, *T. castaneum*, *Onthophagus taurus*, *P. pectoral*, and *Agrilus planipennis*, and the lepidopteran insect *Danaus plexippus* was used to determine the root of the tree. All proteins were pooled together, and OrthoMCL [43] was used for orthologue group identification. The results indicated that 76,623 orthologue groups were identified, and 13,627 gene families were specific to *P. brevitarsis*. Moreover, 2,354 orthologue groups, which were identified as single copy genes that were shared between these species, were selected for subsequence analyses. The selected proteins from these species were concatenated and subjected to multiple alignment using MAFFT [44] and profile-trimming with TrimAI [45]. After that,

BEAST 2 [46] was used to conduct phylogenetic analyses. The phylogenetic tree indicated that *P. brevitarsis* was closely related to *O. taurus*, and the estimated divergence time was around 140 million years ago (Mya) (**Fig. 3**).

Discussion

Scarabaeoidea is a diverse lineage of predominantly plant- and dung-feeding beetles that consists of more than 31,000 described species [47]. The genome of the dung-feeding scarab, *O. taurus*, was sequenced as a part of the i5k project [48]. In this study, we sequenced the genome of *P. brevitarsis*, and this represents the first high quality genome of a plant-feeding scarab. Plant- and dung-feeding scarab beetles are considered sister lineages [49], and they exhibit modes that can be used to test hypotheses of species diversification that may have been driven by interactions with angiosperm and mammal lineages. Therefore, *P. brevitarsis* genomic data could provide useful resources for studies that examine the evolution of insect lineages and major biotic changes in Earth's history. Furthermore, this high-quality reference genome will contribute to research associated with several recent investigations regarding *P. brevitarsis*' harmfulness to crops, usefulness in agricultural waste utilization, edibleness, medicinal value, and applications to insect immunology research.

Availability of supporting data

Raw sequencing reads have been deposited in the Sequence Read Archive (SRA) database with NCBI Bioproject ID PRJNA477715 and PRJNA482477. The genome assembly, gene models and other supporting data, are available via the GigaScience

database GigaDB.

Additional files

Table S1. Estimation of genome characteristics based on 17-mer analysis.

Abbreviations

Gb: gigabases; bp: base pair; kb: kilobases; Mb: megabases; SMRT: single molecule real time; LIS: longest increasing subset algorithm; ASs: allele sequences; HGCs: haploid genome contigs; STAR: Spliced Transcripts Alignment to a Reference; BUSCO: Benchmarking Universal Single-Copy Orthologs; LTR: long terminal repeats; TE: transposable element; LINE: long interspersed nuclear elements; SINE: short interspersed nuclear elements; SRA: sequence read archive; Mya: million years ago.

Competing interests

The authors declare that they have no competing interests.

Author contributions

C.S. and J.Z. designed the study; C.L. and Q.W. collected samples; C.L. and J.Y. and L.G. extracted DNA and RNA samples; Y.G. and P.L. worked on sequencing; C.S., P.L. and K.W. worked on the genome assembly, assessment and annotation; C.S. and K.W. wrote the manuscript. All authors read and approved the final version of the manuscript.

Acknowledgements

This study was supported by the National Key Research and Development Program of China (2017YFD0201204 and 2017YFD0200604) and National Natural Science

Foundation of China (Nos. 31530095 and 41701298).

References

1. Suo Z, Bai M, Li S, Yang H, Li T, Ma D. A geometric morphometric analysis of the morphological variations among Chinese populations of *Protaetia brevitarsis* (Coleoptera: Scarabaeidae) with an inference of the invading source of its Xinjiang populations. Acta Entomol Sin. 2015;58:408–18.
2. Li T, Ma D, Qiang S, Wang X, Wei Y. A study on hosts and the occurrence regularity of *Postosia brevitarsis* Leiwis in west suburb of Urumqi. Xinjiang Agric Sci. 2010;47:320–4.
3. Tian X, Song F, Zhang J, Liu R, Zhang X, Duan J, Shu C. Diversity of gut bacteria in larval *Protaetia brevitarsis* (Coleoptera: Scarabaedia) fed on corn stalk. Acta Entomol Sin. 2017;60:632–41.
4. Ghosh S, Lee S, Jung C, Meyer–Rochow VB. Nutritional composition of five commercial edible insects in South Korea. J Asia Pac Entomol. 2017;20:686–94.
5. Kim S, Weaver CM, Choi M. Proximate composition and mineral content of five edible insects consumed in Korea. CyTA-J Food. 2017;15:143–46.
6. Lee J, Bang K, Hwang S, Cho S. cDNA cloning and molecular characterization of a defensin–like antimicrobial peptide from larvae of *Protaetia brevitarsis seulensis* (Kolbe). Mol BioL Rep. 2016;43:371–79.
7. Lee J, Jo D, Lee A, Park H, Youn K, Yun E, Hwang J, Jun M, Kang B. Hepatoprotective and antineoplastic properties of *Protaetia brevitarsis* larvae. Entomol Res. 2014;44:244–53.
8. Yoo Y, Shin B, Hong J, Lee J, Chee H, Song K, Lee K. Isolation of fatty acids with anticancer activity from *Protaetia brevitarsis* larva. Arch Pharm Res. 2007;30:361–65.
9. Lee J, Lee W, Kim MA, Hwang JS, Na M, Bae JS. Inhibition of platelet aggregation and thrombosis by indole alkaloids isolated from the edible insect *Protaetia brevitarsis seulensis* (Kolbe). J Cell Mol Med. 2016;21:1217-27.
10. Bang K, Hwang S, Lee J, Cho S. Identification of immunity-related genes in the larvae of *Protaetia brevitarsis seulensis* (Coleoptera: Cetoniidae) by a next-generation sequencing-based transcriptome analysis. J Insect Sci. 2015;15:142.
11. Kwon H, Bang K, Cho S. Characterization of the hemocytes in larvae of *Protaetia brevitarsis seulensis*: involvement of granulocyte-mediated phagocytosis. PLoS One. 2014;9(8):e103620.
12. Lee J, Hwang S, Cho S. Immune tolerance to an intestine-adapted bacteria, *Chryseobacterium sp.*, injected into the hemocoel of *Protaetia brevitarsis seulensis*. Sci Rep. 2016;6:31722.
13. Nowoshilow S, Schloissnig S, Fei JF, Dahl A, Pang AWC, Pippel M, Winkler S, Hastie AR, Young G, Roscito JG, Falcon F, Knapp D, Powell S, Cruz A, Cao H, Habermann B, Hiller M, Tanaka EM, Myers EW. The

axolotl genome and the evolution of key tissue formation regulators. Nature. 2018;554:50–5.

14. Delcher A L, Salzberg S L, Phillippy A M. Using MUMmer to Identify Similar Regions in Large Sequence Sets. Current Protocols in Bioinformatics. 2003;10.3.1-10.3.18.
15. Lobo I. Basic Local Alignment Search Tool (BLAST). Journal of Molecular Biology. 2008;215:403-10.
16. Boetzer M, Henkel CV, Jansen HJ, Butler D, Pirovano W. Scaffolding pre-assembled contigs using SSPACE. Bioinformatics. 2011;27:578–9.
17. Walker BJ, Abeel,T, Shea T, Priest M, Abouelliel A, Sakthikumar S, Cuomo CA, Zeng Q, Wortman J, Young SK, Earl AM. Pilon: an integrated tool for comprehensive microbial variant detection and genome assembly improvement. PLoS One. 2014;9:e112963.
18. Li H, Durbin R. Fast and accurate short read alignment with Burrows-Wheeler Transform. Bioinformatics. 2009;25:1754-60.
19. Dobin A1, Davis CA, Schlesinger F, Drenkow J, Zaleski C, Jha S, Batut P, Chaisson M, Gingeras TR. STAR: ultrafast universal RNA-seq aligner. Bioinformatics. 2013;29:15-21.
20. Waterhouse RM, Seppey M, Simao FA, Manni M, Ioannidis P, Klioutchnikov G, Kriventseva EV, Zdobnov EM. BUSCO applications from quality assessments to gene prediction and phylogenomics. Mol Biol Evol. 2017;35:543–8.
21. Benson G. Tandem repeats finder: a program to analyze DNA sequences. Nucleic Acids Res. 1999;27:573–80.
22. Bao W, Kojima KK, Kohany O. Repbase Update: a database of repetitive elements in eukaryotic genomes. Mob DNA. 2015;6:11.
23. Tarailo-Graovac M, Chen N. Using RepeatMasker to identify repetitive elements in genomic sequences. In: Current Protocols in Bioinformatics. John Wiley & Sons; 2009;4.10.1-4.10.14.
24. Xu Z, Wang H. LTR_FINDER: an efficient tool for the prediction of full-length LTR retrotransposons. Nucleic Acids Res. 2007;35(Web Server issue):W265–8.
25. Price AL, Jones NC, Pevzner PA. De novo identification of repeat families in large genomes. Bioinformatics. 2005;21 Suppl 1:i351–8.
26. Lowe TM, Chan PP. tRNAscan-SE On-line: integrating search and context for analysis of transfer RNA genes. Nucleic Acids Res. 2016;44(W1):W54–7.
27. Lagesen K, Hallin P, Rodland EA, Staerfeldt HH, Rognes T, Ussery DW. RNAmmer: consistent and rapid annotation of ribosomal RNA genes. Nucleic Acids Res. 2007;35:3100–8.
28. Daub J, Eberhardt RY, Tate JG, Burge SW. Rfam: annotating families of non-coding RNA sequences. Methods Mol Biol. 2015;1269:349–63.
29. She R, Chu JS, Wang K, Pei J, Chen N. GenBlastA: enabling BLAST to identify homologous gene sequences. Genome Res. 2009;19:143–9.

30. Birney E, Clamp M, Durbin R. GeneWise and Genomewise. Genome Res. 2004;14:88–95.
31. Stanke M, Keller O, Gunduz I, Hayes A, Waack S, Morgenstern B. AUGUSTUS: *ab initio* prediction of alternative transcripts. Nucleic Acids Res. 2006;34(Web Server issue):W435–9.
32. Besemer J, Borodovsky M. GeneMark: web software for gene finding in prokaryotes, eukaryotes and viruses. Nucleic Acids Res 2005;33(Web Server issue):W451–4.
33. Korf I. Gene finding in novel genomes. BMC Bioinformatics. 2004;5:59.
34. Pertea M, Pertea GM, Antonescu CM, Chang TC, Mendell JT, Salzberg SL. StringTie enables improved reconstruction of a transcriptome from RNA-seq reads. Nat Biotechnol. 2015;33:290–5.
35. Haas BJ, Salzberg SL, Zhu W, Pertea M, Allen JE, Orvis J, White O, Buell CR, Wortman JR. Automated eukaryotic gene structure annotation using EVidenceModeler and the Program to Assemble Spliced Alignments. Genome Biol. 2008;9:R7.
36. Kanehisa M, Goto S. KEGG: Kyoto encyclopedia of genes and genomes. Nucleic Acids Res. 2000;28:27–30.
37. Boeckmann B, Bairoch A, Apweiler R, Blatter MC, Estreicher A, Gasteiger E, Martin MJ, Michoud K, O'Donovan C, Phan I, Pilbout S, Schneider M. The SWISS-PROT protein knowledgebase and its supplement TrEMBL in 2003. Nucleic Acids Res. 2003;31:365–70.
38. Quevillon E, Silventoinen V, Pillai S, Harte N, Mulder N, Apweiler R, Lopez R. InterProScan: protein domains identifier. Nucleic Acids Res. 2005;33(Web Server issue):W116–20.
39. Mistry J, Finn R. Pfam: a domain-centric method for analyzing proteins and proteomes. Methods Mol Biol. 2007;396:43–58.
40. Letunic I, Copley RR, Schmidt S, Ciccarelli FD, Doerks T, Schultz J, Ponting CP, Bork P. SMART 4.0: towards genomic data integration. Nucleic Acids Res. 2004;32(Database issue):D142–4.
41. Mi H, Huang X, Muruganujan A, Tang H, Mills C, Kang D, Thomas PD. PANTHER version 11: expanded annotation data from Gene Ontology and Reactome pathways, and data analysis tool enhancements. Nucleic Acids Res. 2017;45:D183–9.
42. Sigrist CJ, Cerutti L, de Castro E, Langendijk-Genevaux PS, Bulliard V, Bairoch A, Hulo N. PROSITE, a protein domain database for functional characterization and annotation. Nucleic Acids Res. 2010;38(Database issue):D161–6.
43. Li L, Stoeckert CJ Jr, Roos DS. OrthoMCL: identification of ortholog groups for eukaryotic genomes. Genome Res. 2003;13:2178–89.
44. Katoh K, Standley DM. MAFFT multiple sequence alignment software version 7: improvements in performance and usability. Mol Biol Evol. 2013;30:772–80.
45. Capella-Gutierrez S, Silla-Martinez JM, Gabaldon T. trimAl: a tool for

automated alignment trimming in large-scale phylogenetic analyses. Bioinformatics. 2009;25:1972–3.

46. Bouckaert R, Heled J, Kuhnert D, Vaughan T, Wu CH, Xie D, Suchard MA, Rambaut A, Drummond AJ. BEAST 2: a software platform for Bayesian evolutionary analysis. PLoS Comput Biol. 2014;10:e1003537.
47. Mckenna DD, Wild AL, Kanda K, Bellamy CL, Beutel RG, Caterino MS, Farnum CW, Hawks DC, Ivie MA, Jameson ML, Leschen RA. The beetle tree of life reveals that Coleoptera survived end - Permian mass extinction to diversify during the Cretaceous terrestrial revolution. Syst Entomol. 2015;40:835–880.
48. McKenna DD, Scully ED, Pauchet Y, Hoover K, Kirsch R, Geib SM, Mitchell RF, Waterhouse RM, Ahn SJ, Arsala D, Benoit JB, Blackmon H, Bledsoe T, Bowsher JH, Busch A, Calla B, Chao H, Childers AK, Childers C, Clarke DJ, Cohen L, Demuth JP, Dinh H, Doddapaneni H, Dolan A, Duan JJ, Dugan S, Friedrich M, Glastad KM, Goodisman MA, Haddad S, Han Y, Hughes DS, Ioannidis P, Johnston JS, Jones JW, Kuhn LA, Lance DR, Lee CY, Lee SL, Lin H, Lynch JA, Moczek AP, Murali SC, Muzny DM, Nelson DR, Palli SR, Panfilio KA, Pers D, Poelchau MF, Quan H, Qu J, Ray AM, Rinehart JP, Robertson HM, Roehrdanz R, Rosendale AJ, Shin S, Silva C, Torson AS, Jentzsch IM, Werren JH, Worley KC, Yocum G, Zdobnov EM, Gibbs RA, Richards S. Genome of the Asian longhorned beetle (Anoplophora glabripennis): a globally significant invasive species, reveals key functional and evolutionary innovations at the beetle-plant interface. Genome Biol. 2016;17(1):227.
49. Browne J, Scholtz CH. A phylogeny of the families of Scarabaeoidea (Coleoptera). Syst Entomol. 1999;24:51–84.
50. Marçais G and Kingsford C. A fast, lock-free approach for efficient parallel counting of occurrences of k-mers. Bioinformatics. 2011;27(6):764.

Tables

Table 1. Summary statistics of generated sequence data

Library Name	Experiment Title	Sequencing Instrument	Total Bases (bp)	Accession No.
Raw_200_DNA_Hiseq	DNA PE library	Illumina HiSeq 2500	48,637,157,380	-
Raw_420_DNA_Hiseq	DNA PE library	Illumina HiSeq 2500	59,133,181,272	-
Filtered_200_DNA_Hiseq	DNA PE library	Illumina HiSeq 2500	46,322,512,285	SRR7421508
Filtered_420_DNA_Hiseq	DNA PE library	Illumina HiSeq 2500	40,349,624,172	SRR7421507
DNA_PacBio1	DNA PacBio library	PacBio RS II	1,248,598,019	SRR7429397
DNA_PacBio2	DNA PacBio library	PacBio RS II	1,742,919,487	SRR7429396
DNA_PacBio3	DNA PacBio library	PacBio RS II	1,471,376,296	SRR7429395
DNA_PacBio4	DNA PacBio library	PacBio RS II	1,446,032,590	SRR7429394
DNA_PacBio5	DNA PacBio library	PacBio RS II	1,410,533,432	SRR7429401
DNA_PacBio6	DNA PacBio library	PacBio RS II	1,303,543,797	SRR7429400
DNA_PacBio7	DNA PacBio library	PacBio RS II	1,185,731,970	SRR7429399
DNA_PacBio8	DNA PacBio library	PacBio RS II	1,360,241,545	SRR7429398
DNA_PacBio9	DNA PacBio library	PacBio RS II	1,033,036,210	SRR7429403
DNA_PacBio10	DNA PacBio library	PacBio RS II	981,818,132	SRR7429402
DNA_PacBio11	DNA PacBio library	PacBio RS II	1,192,589,806	SRR7429389
DNA_PacBio12	DNA PacBio library	PacBio RS II	707,437,407	SRR7429388
DNA_PacBio13	DNA PacBio library	PacBio RS II	659,418,664	SRR7429391
DNA_PacBio14	DNA PacBio library	PacBio RS II	618,638,129	SRR7429390
DNA_PacBio15	DNA PacBio library	PacBio RS II	630,384,409	SRR7429393
DNA_PacBio16	DNA PacBio library	PacBio RS II	761,167,622	SRR7429392
DNA_PacBio17	DNA PacBio library	PacBio RS II	2,180,394,708	SRR7470031
DNA_PacBio18	DNA PacBio library	PacBio RS II	2,035,388,872	SRR7470028
DNA_PacBio19	DNA PacBio library	PacBio RS II	1,796,143,706	SRR7470027
DNA_PacBio20	DNA PacBio library	PacBio RS II	1,980,034,243	SRR7470030
DNA_PacBio21	DNA PacBio library	PacBio RS II	2,229,575,050	SRR7470029
Egg	RNA-Seq library	Illumina HiSeq 2500	6,049,557,600	SRR7418793
Larva	RNA-Seq library	Illumina HiSeq 2500	6,112,599,900	SRR7418797
Pre-pupal	RNA-Seq library	Illumina HiSeq 2500	6,168,021,600	SRR7418791
Middle pupal	RNA-Seq library	Illumina HiSeq 2500	6,015,743,700	SRR7418789
Late pupal	RNA-Seq library	Illumina HiSeq 2500	6,260,516,400	SRR7418796
Male adult	RNA-Seq library	Illumina HiSeq 2500	6,054,195,300	SRR7418798
Female adult	RNA-Seq library	Illumina HiSeq 2500	6,188,099,400	SRR7418790
Forewing (D1)	RNA-Seq library	Illumina HiSeq 2500	6,234,580,800	SRR7585362
Forewing (D3)	RNA-Seq library	Illumina HiSeq 2500	6,208,411,800	SRR7418792
Underwing (D1)	RNA-Seq library	Illumina HiSeq 2500	6,154,223,400	SRR7418801
Underwing (D3)	RNA-Seq library	Illumina HiSeq 2500	6,172,792,500	SRR7418794
Head (D1)	RNA-Seq library	Illumina HiSeq 2500	6,090,345,900	SRR7418799
Head (D3)	RNA-Seq library	Illumina HiSeq 2500	6,247,745,100	SRR7418800

Note: D1 or D3, tissues (forewing, underwing, and head) of newly (one-day) or three-day emerged adults.

Table 2. Summary statistics of data during the assembly process

	Number	Total bases (bp)	N50	Average length (bp)
Filtered PacBio reads	1,353,926	14,251,368,546	16,059	10,525
Elementary contigs	8,760	1,127,134,570	190,967	128,668
HGCs	3,816	738,878,186	347,620	193,626
ASs	4,939	391,445,919	91,687	79,256
Scaffolds	313	751,076,257	2,939,522	2,399,604
Corrected HGCs	3,821	739,117,100	327,214	193,435
Corrected ASs	4,939	393,190,609	92,105	79,609
Corrected scaffolds	313	751,076,257	2,939,522	2,399,604

Table 3. Summary statistics of Illumina genome sequencing reads mapped onto the assemblies

	Average depth	Lowest depth	Highest depth
Corrected HGCs	121.9	73.24	167.07
Corrected ASs	85.63	13.08	1221.48
Corrected scaffolds	122.2	73.24	167.07

Table 4. Summary statistics of RNA-seq reads mapped onto the assemblies

Sample	No. of reads	Mapped to scaffolds		Mapped to allele sequences	
		No. of mapped reads	Percentages	No. of mapped reads	Percentages
Egg	40,330,384	40,330,384	100.00%	14,961,772	37.10%
Larva	40,750,666	40,750,666	100.00%	15,266,678	37.46%
Pre-pupal stage	41,120,144	41,120,144	100.00%	15,172,926	36.90%
Middle pupal stage	40,104,958	40,104,958	100.00%	15,307,418	38.17%
Late pupal stage	41,736,776	41,736,776	100.00%	17,178,294	41.16%
Male adult	40,361,302	40,361,302	100.00%	14,518,842	35.97%
Female adult	41,253,996	41,253,996	100.00%	16,135,954	39.11%
Forewing (D1)	41,563,872	41,563,872	100.00%	9,233,440	22.22%
Forewing (D3)	41,389,412	41,389,412	100.00%	13,516,032	32.66%
Underwing (D1)	41,028,156	41,028,156	100.00%	14,943,970	36.42%
Underwing (D3)	41,151,950	41,151,950	100.00%	16,851,062	40.95%
Head (D1)	40,602,306	40,602,306	100.00%	11,935,160	29.40%

Head (D3)	41,651,634	41,651,634	100.00%	11,779,198	28.28%

Note: D1 or D3, tissues (forewing, underwing, and head) of newly (one-day) or three-day emerged adults.

Table 5. Benchmarking Universal Single-copy Orthologs found in Coleopteran genomes

Status	Complete	Fragment	Missing	Duplication
O. Taurus	80.45%	10.00%	9.55%	8.80%
D. ponderosae	81.47%	8.53%	10.00%	10.87%
A. glabripennis	82.31%	8.70%	8.99%	9.12%
A. planipennis	91.90%	2.70%	5.40%	4.10%
P. brevitarsis	93.00%	1.90%	5.10%	7.20%
T. castaneum	96.59%	2.90%	0.51%	9.40%
P. pectoral	98.80%	0.60%	0.60%	7.20%

Table 6. Summary of identified repeat elements in the *P. brevitarsis* genome

Repeat element	Repeat elements from haploid genome		Repeat elements from allele sequences	
	Length (bp)	Percentage (%)	Length (bp)	Percentage (%)
LTR	109,722,085	14.35	60,133,491	15.29
LINE	101,529,627	13.28	52,758,849	13.42
SINE	259,936	0.03	50,366	0.01
DNA element	166,788,392	21.81	92,972,801	23.65
Simple repeat	4,749,908	0.62	2,485,661	0.63
Low complexity	1,132,919	0.15	656,626	0.17
RC	7162276	0.94	5,220,692	1.33
Satellite	304734	0.04	221,437	0.06
Other	131,605	0.02	99,113	0.03
Unclassified	4,451,277	0.58	5,618,712	1.43
Total	396,232,759	51.82	220,217,748	56.02

Table 7. Summary of annotated genes in the *P. brevitarsis* genome

	**Genes from haploid genome**		**Genes from allele sequences**	
	**No. of annotated genes**	**Percentage (%)**	**No. of annotated genes**	**Percentage (%)**
KEGG	15,828	71.16%	7,980	67.17%
Swiss-Prot	10,509	47.25%	5,179	43.59%
Nr	17,487	78.62%	8,757	73.71%
Nt	3,688	16.58%	1,855	15.61%
TrEMBL_eggNOG	15,986	71.87%	8,029	67.58%
No. of total annotated gene	17,625	79.24%	8,887	74.80%

Figures:

Fig. 1 Image of adult of the white-spotted flower chafer, *Protaetia brevitarsis*.

Fig. 2 The 17-mer distribution of the *P. brevitarsis* genome using the jellyfish [50] program with 420-bp paired-end whole genome sequencing data.

Fig. 3 Phylogenetic relationships of *P. brevitarsis* and six Coleoptera insects based on 2,354 orthologue genes. Estimated divergence times using *D. ponderosae-T. castaneum* [180 Mya] as the calibration time are shown [47].

 Click here to download Figure Figures.docx

Fig. 1

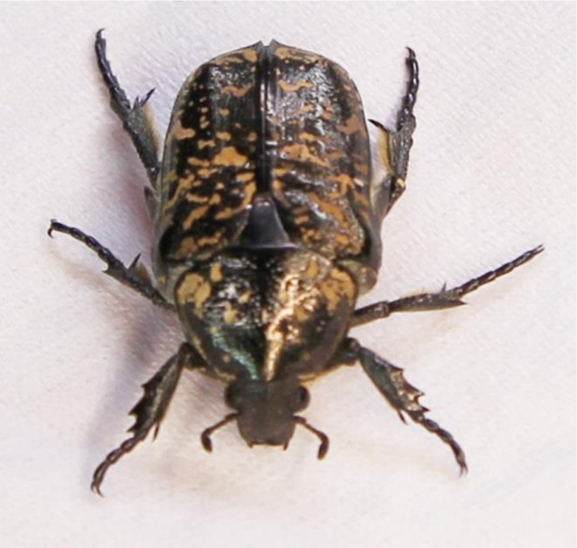

Fig. 2

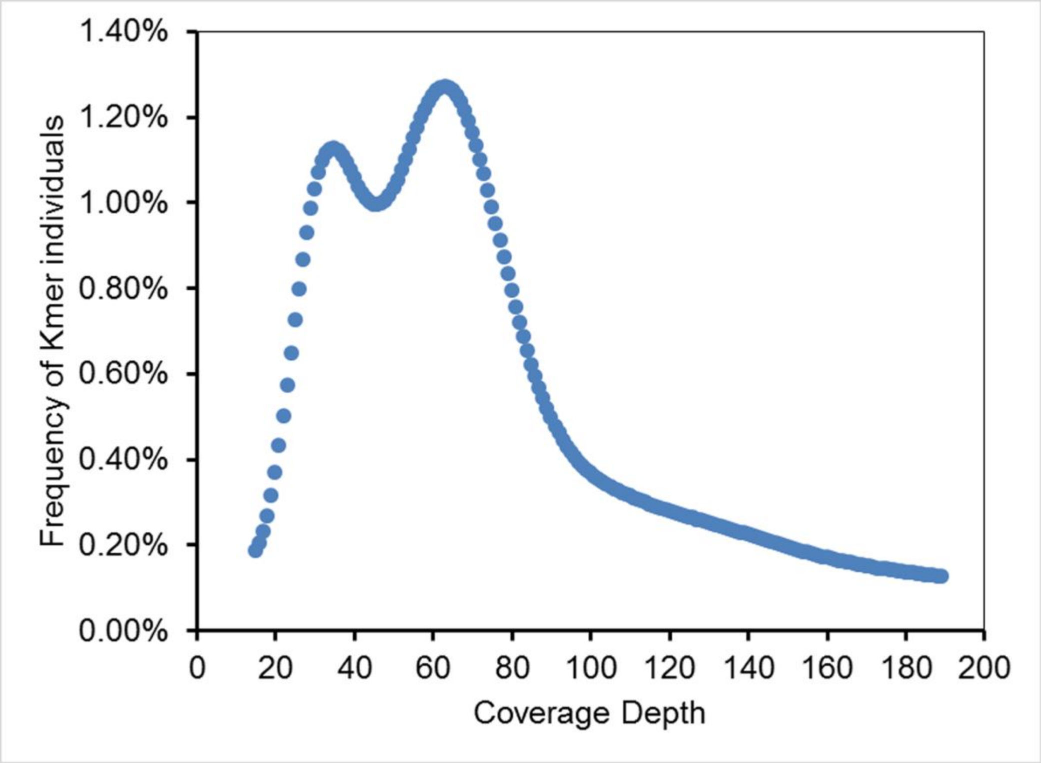

Fig. 3

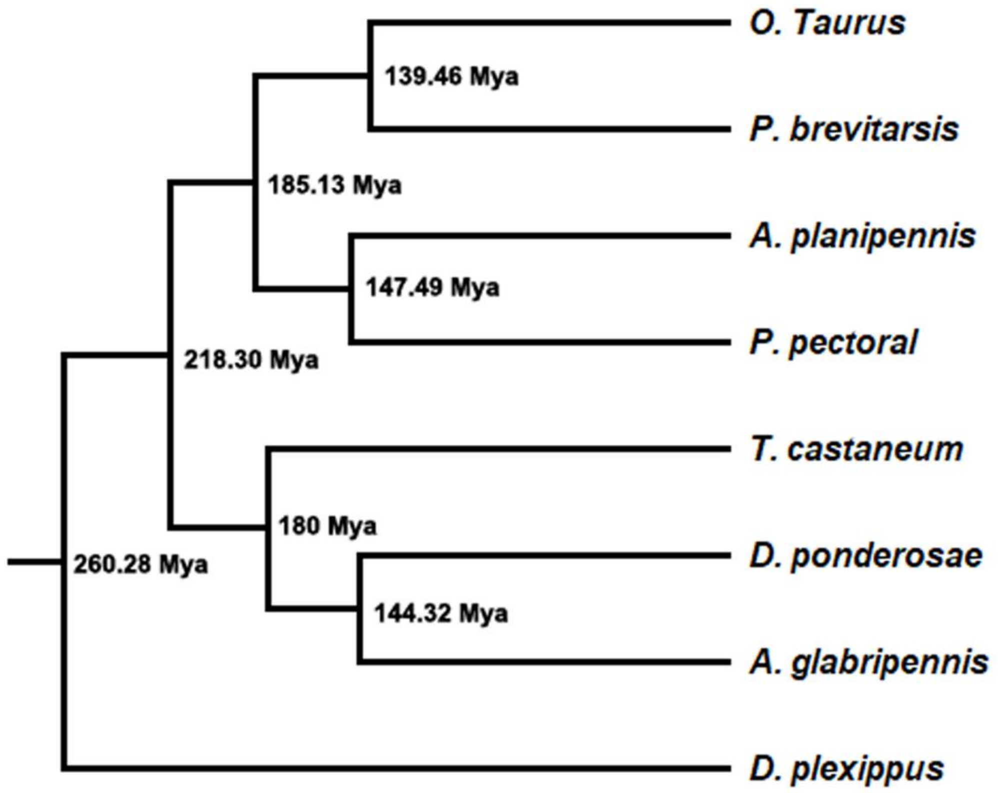
O. Taurus
P. brevitarsis
A. planipennis
P. pectoral
T. castaneum
D. ponderosae
A. glabripennis
D. plexippus
139.46 Mya
185.13 Mya
147.49 Mya
218.30 Mya
180 Mya
260.28 Mya
144.32 Mya

table s1

Click here to access/download
Supplementary Material
Table S1.docx
