# Supplementary material for: De novo genome assembly of the white-spotted flower chafer (Protaetia brevitarsis)
Source: Gigascience. 2019 Apr 5;8(4):giz019. doi: 10.1093/gigascience/giz019 (PMC6449472; doi:10.1093/gigascience/giz019)
Supplement: Supplemental File [file giz019_supplemental_file.docx]

**Table S1 Estimation of genome characteristics based on 17-mer analysis.**

| K-mer value | Peakmain | Amount of error k-mer | Amount of total k-mer | Amount of used k-mer | GenomeSize (bp) | Heterozygosity rate (%) | Repeat Rate (%) |
| --- | --- | --- | --- | --- | --- | --- | --- |
| 17 | 63 | 9,831,740,142 | 60,101,962,676 | 50,270,222,534 | 810,810,040 | 2.35 | 18.29 |

**Table S2 The software used in the study.**

| Software | Version | Function | Key parameters | Web resource |
| --- | --- | --- | --- | --- |
| Data QC | | | | |
| bash5tools | V1.0 | Pacbio RSII data quality control | bash5tools.py --minReadScore 0.80 | https://github.com/PacificBiosciences/pbh5tools |
| AdapterTrim | In house script | Illumina data adapter filter | AdapterTrim.exe -a adapter.fasta -i ./ | https://github.com/lpp1985/lpp_Script/blob/master/go/src/AdapterTrim.go |
| Quality_Stats | In house script | Illumina data quality control | quality_stats_Pairend_Distri_33.py -v 2 -t -q 20 -r 0.2 | <https://github.com/lpp1985/lpp_Script/blob/master/quality_stats_Pairend_Distri_33.py> |
| Genome Survey | | | | |
| Jellyfish | V2.2.6 | Kmer Counting | jellyfish count  -m 17&&  jellyfish  histo  -l 2 -h 255 -t 64 -o kmer_freq.stats kmer | https://www.cbcb.umd.edu/software/jellyfish/ |
| Genome Assembly | | | | |
| MARVEL | ba5a9d4 | Genome assembly | Coverage=38 | https://github.com/schloi/MARVEL |
| Blasr | V2.0.0 | Filter mitochondrial genome reads | Blasr -maxScore -2000 -sam | https://github.com/PacificBiosciences/blasr |
| Canu | V1.6 | Mitochondrial genome assembly | Canu genomesize=25k | https://canu.readthedocs.io/en/latest/ |
| Mummer | V3.1 | Allele alignment | nucmer --mumreference -b 500 -g 200 -l 100 -p Ref &&delta-filter -1 -l 1000 -m Ref.delta >Filter.delta &&show-coords -roTlH Filter.delta\|SortMummer_Align.py \| less >AlignHSP.tsv | http://mummer.sourceforge.net/ |
| SSPACE | Longread V.-1-1 | Scaffolding pre-assembled contigs | Default parameter | www.baseclear.com/bioinformatics-tools |
| Blast_Chain | In house script | Cluster all blast HSPs into LIS chain and then calculate whether its coverage achieve 85%. | cat blast.out\|Blast_Chain 0.85 | https://github.com/lpp1985/lpp_Script/blob/master/go/src/Blast_Chain.go |
| Pilon | V1.16 | Contig Polishing | Default parameter | http://software.broadinstitute.org/software/pilon/ |
| BWA | V0.7.17 | Mapping reads to assemblies. | Default parameter | http://bio-bwa.sourceforge.net/ |
| RNA-Seq Alignment | | | | |
| STAR | V20201 | RNA-Seq Mapping | STAR --outSAMtype BAM SortedByCoordinate --outFilterType BySJout --outFilterMultimapNmax 20 --alignSJoverhangMin 8 --alignSJDBoverhangMin 1 --outSAMunmapped Within --outFilterMismatchNmax 20 --outFilterMismatchNoverLmax 0.04 --alignIntronMin 20 --alignIntronMax 10000 --alignMatesGapMax 10000 --chimSegmentMin 20 --twopassMode Basic --outSAMstrandField intronMotif RemoveNoncanonical --alignTranscriptsPerReadNmax  10000 | <https://github.com/alexdobin/STAR> |
| Completeness Validataion | | | | |
| BUSCO | V1.1b1 | Compleness validation | run_BUSCO.py --lineage_path insecta_odb9 --mode genome | https://busco.ezlab.org/ |
| bamtools | V2.4.1 | Calcate RNA-Seq alignment situation | bamtools stats | https://bioinformatics.readthedocs.io/en/latest/bamtools/ |
| Genome annotation | | | | |
| RepeatModeler | open-1.0.8 | RepeatMasking | Default parameter | http://www.repeatmasker.org/RepeatModeler/ |
| TRF | V. 4.07b | Tandem Repeat Masking | Default parameter | https://tandem.bu.edu/trf/trf.html |
| RepeatMasker | open-4.0.7 | RepeatMasking | RepeatMasker -e ncbi -pa 8 | http://www.repeatmasker.org/ |
| LTR_Finder | V. 1.0.5 | RepeatMasking | ltr_finder -w 2 scaff.fa | http://tlife.fudan.edu.cn/tlife/ltr_finder/ |
| RepeatScout | V1.0.5 | RepeatMasking | RepeatScout -sequence Scaffolds.fa | https://bix.ucsd.edu/repeatscout/ |
| RepeatProteinMask | V. 1.36 | RepeatMasking | Default parameter | http://www.repeatmasker.org/ |
| tRNAscan-SE | V1.3.1 | tRNA prediction | Default parameter | http://lowelab.ucsc.edu/tRNAscan-SE/ |
| RNAmmer | V1.2 | rRNA prediction | rnammer  -S euk -m tsu,ssu,lsu | http://www.cbs.dtu.dk/services/RNAmmer/ |
| GenBlastA | V1.0.1 | Precompute candidate region of gene coding | genblasta -P blast -pg tblastn -p T | http://genome.sfu.ca/genblast/a.html |
| GeneWise | wise2-4-1 | Align cDNA to genome | genwise -gff -sum | https://www.ebi.ac.uk/Tools/psa/genewise/ |
| Augustus | V3.3 | Gene Prediction | python -m jcvi.annotation.train augustus Baixing complete_Longest_isoform.gff3 Scaffolds.fa &&gff2gbSmallDNA.pl complete_Longest_isoform.gff3 Assembly.fasta 1000 raw.gb &&etraining --species=Bai raw.gb 2> train.err &&augustus --species=Niao training.gb --gff3=on --UTR=off | http://augustus.gobics.de/ |
| SNAP | V1.0 | Gene Prediction | python -m jcvi.annotation.train snap Bai genemark.good.gff3  AssenblyEND.fa  --maker_home=maker &&./snap -quiet -gff HMM/thale DNA/Bai.dna.gz | https://github.com/KorfLab/SNAP |
| Genemarks | V1.0 | Gene Prediction | gmes_petap.pl --ET --sequence seq.fna --ET introns.gff --et_score 10 | http://exon.gatech.edu/GeneMark/ |
| StringTie | V1.3.3b | cDNA prediction | Default parameter | http://ccb.jhu.edu/software/stringtie/ |
| Evidence Modeler | [V1.1.1](https://github.com/EVidenceModeler/EVidenceModeler/releases/tag/v1.1.1) | Gene Prediction result integration | partition_EVM_inputs.pl --genome --gene_predictions GenePrediction.gff3 --transcript_alignments stringtie.gff3 --protein_alignments genewise.gff --repeats Repeat.gff3 --segmentSize 1000000 --overlapSize 10000 --partition_listing partitions_list.out &  && create_weights_file.pl -A GenePrediction.gff3 -P genewise.gff -T Mian.pasa_assemblies.gff3 >weights3.txt&&write_EVM_commands.pl --weights `pwd`/weights.txt --genome --gene_predictions `pwd`/GenePrediction.gff3 --transcript_alignments `pwd`/stringtie.gff3 --protein_alignments `pwd`/genewise.gff --repeats Repeat.gff3 --output_file_name evm.out --partitions `pwd`/partitions_list.out > commands.list &&nohup cat commands.list && convert_EVM_outputs_to_GFF3.pl --partitions partitions_list.out --output filter.out --genome ../Scaffolds.fa | https://evidencemodeler.github.io/ |
| Blast | V2.2.17 | Function annotation | blastn -outfmt 6-evalue 1e-5 | https://blast.ncbi.nlm.nih.gov/Blast.cgi?CMD=Web&PAGE_TYPE=BlastHome |
| Phylogentics Analysis | | | | |
| OrthoMCL | V2.0.9 | Orthologue gene prediction | orthomclFilterFasta fasta/ 10 20  makeblastdb -in goodProteins.fasta -dbtype prot blast.sh goodProteins.fasta orthomclBlastParser align.out fasta/ >similarSequences.txt orthomclLoadBlast orthomcl.config.template similarSequences.txt orthomclPairs orthomcl.config.template run.log cleanup=yes orthomclDumpPairsFiles orthomcl.config.template  mcl mclInput --abc -I 1.5 -o mclOutput  orthomclMclToGroups Family 0001 < mclOutput > groups.txt GetSingleOrtholog.py Ortholog.list groups.txt | http://orthomcl.org/orthomcl/ |
| Beast2 | V2 | Phylogenetic analysis of molecular sequences. | Default parameter | https://github.com/CompEvol/beast2 |
| MAFFT | V7.271 | Multiple alignment of ortholog gene | mafft --clustalout | https://mafft.cbrc.jp/alignment/software/ |
| TrimAI | V2.1 | Alignment profile triming | Default parameter | http://trimal.cgenomics.org/ |
