# Supplementary material for: De novo genome assembly of the white-spotted flower chafer (Protaetia brevitarsis)
Source: Gigascience. 2019 Apr 5;8(4):giz019. doi: 10.1093/gigascience/giz019 (PMC6449472; doi:10.1093/gigascience/giz019)
Supplement: Response_to_Reviewer_Comments_Original_Submission.pdf [file giz019_response_to_reviewer_comments_original_submission.pdf]

Author's Response To Reviewer Comments

Close

Dear editor,
I would like to thank the Reviewers for their comments concerning my manuscript. According to the suggestions, I have made the following changes:
Firstly, in this revision, we update the assembly by remove the contaminations, add the mitochondrial genome sequence. The updated assembly have been deposited in NCBI Genomes with accession number RXPK00000000. Therefore, the related data also revised in the paper. All revised text make as color blue.
We also response to reviews in the personal cover.

Sincerely,

Changlong Shu
Associate professor
Institute of Plant Protection, Chinese Academy of Agricultural Sciences
No. 2 Yuan-ming-yuan West Road, Haidian District
Beijing 100193
P. R. China
Email : clshu@ippcaas.cn
FAX/TEL : 010-62812642

Close
