# Supplementary material for: De novo genome assembly of the white-spotted flower chafer (Protaetia brevitarsis)
Source: Gigascience. 2019 Apr 5;8(4):giz019. doi: 10.1093/gigascience/giz019 (PMC6449472; doi:10.1093/gigascience/giz019)
Supplement: Reviewer_1_Report_Original_Submission -- Eduardo Enrique Zattara, Ph.D. — 8/26/2018 Reviewed [file giz019_reviewer_1_report_original_submission.pdf]

Reviewer Report

Title: De novo genome assembly of the white-spotted flower chafer (Protaetia brevitarsis)

Version: Original Submission **Date:** 8/26/2018

Reviewer name: Eduardo Enrique Zattara, Ph.D.

Reviewer Comments to Author:

In this data note, Shu and colleagues present the results of their efforts to sequence and assemble the genome of the scarab beetle Protaetia brevitarsis, an insect of interest to both basic and applied research. After adequately justifying the relevance of this species as a model for study, they present a detailed report of how they sequenced and assembled its genome, including dealing with high levels of heterozygosity, and then show the results of their efforts to annotate the assembly. They use a number of standard validation tools to show the quality of their assembly, and briefly discuss the potential uses of this dataset in future research. The overall quality of the genome assembly is great, and the authors take advantage of the data note format offered in the journal to extensively describe the assembly and annotation process, without trying to extract other unsupported conclusions. I must also commend the authors for the good quality of writing, given only a few typos and minor grammar issues are to be found. I find this manuscript is in great shape, a good fit for this journal, and recommend its publication.
I only have a few comments that the authors should consider, that follow:
Lines 112-146: I am not sure I fully understood the strategy used to detect and annotate alleles. Since no reference is given, I assume anyone wanting to use the same strategy to assemble other genomes should be able to get all the necessary information from this paper. Thus, it might be worthwhile to consider adding an figure helping to explain the process, or if such information can be found elsewhere, a reference to it.
Lines 155-157: I was surprised that all (=100%) RNA-seq reads mapped to the assembled reference. I do not have experience using STAR, but using other mappers I never got fewer than ~5% reads not mapping, even when using a reference assemble from reads. I do not say 100% mapping is not possible, but I suggest the authors double-check this result.
Line 209: Does the number of genes (22242) include non-coding genes like tRNAs, miRNAs, etc.? Please, be explicit about it, as often times genome annotations do not include non-protein coding genes.
Line 224: Do not abbreviate the genera, unless it has already been mentioned in the same paragraph or at least section.
Lines 223-225: Please, include NCBI accession numbers for the genome assemblies used for this analyses, as there might be more than one version of the assembly.
Lines 225-226: Where it says "was used to determine the root of the tree" it should say "was used to root the tree".
Lines 238-239: The reference given is the genome report for the Asian longhorn beetle. Authors should instead cite the i5k assembly for Onthophagus taurus found at http://dx.doi.org/10.15482/USDA.ADC/1255156 . As for the i5k pilot project, a recent manuscript summarizing its main findings is currently available as a preprint at

https://www.biorxiv.org/node/116903.full and represent a more appropriate citation. I also recommend the authors to take a look at this important paper as it might contain some interesting insights and context to their manuscript.
Table 5, Figure 3 and elsewhere: the species part of the binomen of Onthophagus taurus should not be capitalized. Also, genus should be spelled in full here.

Level of Interest

Please indicate how interesting you found the manuscript: Choose an item.

Quality of Written English

Please indicate the quality of language in the manuscript: Choose an item.

Declaration of Competing Interests

Please complete a declaration of competing interests, considering the following questions:

- Have you in the past five years received reimbursements, fees, funding, or salary from an organisation that may in any way gain or lose financially from the publication of this manuscript, either now or in the future?
- Do you hold any stocks or shares in an organisation that may in any way gain or lose financially from the publication of this manuscript, either now or in the future?
- Do you hold or are you currently applying for any patents relating to the content of the manuscript?
- Have you received reimbursements, fees, funding, or salary from an organization that holds or has applied for patents relating to the content of the manuscript?
- Do you have any other financial competing interests?
- Do you have any non-financial competing interests in relation to this paper?

If you can answer no to all of the above, write 'I declare that I have no competing interests' below. If your reply is yes to any, please give details below.

I declare that I have no competing interests

I agree to the open peer review policy of the journal. I understand that my name will be included on my report to the authors and, if the manuscript is accepted for publication, my named report including any attachments I upload will be posted on the website along with the authors' responses. I agree for my report to be made available under an Open Access Creative Commons CC-BY license (http://creativecommons.org/licenses/by/4.0/). I understand that any comments which I do not wish to be included in my named report can be included as confidential comments to the editors, which will not be published.

I agree to the open peer review policy of the journal

To further support our reviewers, we have joined with Publons, where you can gain additional credit to further highlight your hard work (see: https://publons.com/journal/530/gigascience). On publication of this paper, your review will be automatically added to Publons, you can then choose whether or not to claim your Publons credit. I understand this statement.

Yes Choose an item.
