# Supplementary material for: De novo genome assembly of the white-spotted flower chafer (Protaetia brevitarsis)
Source: Gigascience. 2019 Apr 5;8(4):giz019. doi: 10.1093/gigascience/giz019 (PMC6449472; doi:10.1093/gigascience/giz019)
Supplement: Reviewer_2_Report_Original_Submission -- Bent Petersen — 11/26/2018 Reviewed [file giz019_reviewer_2_report_original_submission.pdf]

Reviewer Report

Title: De novo genome assembly of the white-spotted flower chafer (Protaetia brevitarsis)

Version: Original Submission **Date:** 11/26/2018

Reviewer name: Bent Petersen

Reviewer Comments to Author:

The authors have established a high quality draft genome of the white-spotted flower chafer, Protartia brevitarsis, which is an insect that is distributed in most Asian countries. The published genome will be a useful resource to provide further insight into the biology of this agricultural harmful species. The manuscript is well written, but there is a few places where the language could need a little brush up. Here I am referring to sentences in the manuscript, which is quickly in one sentence writing what will be explained in several sentences just below. This could be re-written to give a better flow.
1. The authors are removing bases that have more than 20% low-quality bases, but they are not writing that they are trimming the low-quality bases, only discarding them. To me that sounds like a read that would have 19% low-quality bases would still be accepted and that should not be the case. Low quality reads should be trimmed from the read in order to call it clean-data.
2. Which quality criteria (Phred score) was used to differentiate a low-quality base from an accepted one?
3. The authors write that they discard reads with more than 10bp overlap with an adapter sequence. Again that does not sound to me like actual adapter removal was performed, but it is still possible to have a small part of an adapter. Proper adapter removal should be performed, and I can recommend the free tool AdapterRemoval (https://bmcresnotes.biomedcentral.com/articles/10.1186/s13104-016-1900-2).
4. The section starting from line 97-103 is called "Genome size and heterozygosity estimation". Nothing is mentioned about heterozygosity in this section and it is not clear how the authors estimated the genome size. This section needs to be expanded.
5. Line 112, Which quality thresholds/settings was used for the quality-filtering?
6. Line 116, I am not sure what you mean by ".. and the average quality was 0.837" => 0.837 what?
7. Line 117, "Marvel (ba5a9d4)", is ba5a9d4 refererring to a specific version?
8. Line 123, Which thresholds was used for BLAST? Default values? Citation to the original publication is missing "https://www.ncbi.nlm.nih.gov/pubmed/2231712"
9. Line 127 - 132. This section is unclear to me. Have you manually looked at dot-plots for all 4,939 allele sequences??
10. Line 239, I don't understand why the authors are writing one sentence about the genome sequencing of O.Taurus and then referring to a publication for the genome of Anoplophora glabripennis. This does not make sense and nothing is explained in relation to why O.Taurus was mentioned.
11. The mitochondrial genome of Protaetia brevitarsis was published in 2013, for some reason the authors decided not to compare their genome to this data. With such a high coverage they should have been able to assembly the mitochondrial genome as well.

The authors are in general not writing which settings/thresholds they have used for most of the used tools, tools that have many parameters to tweak on. If default values were used, please write that, and if not the state the settings that was changed from default.
In general I think the manuscript could use some biological relation to get more interesting. As it is now, it is more or less just a genome announcement, good work though, but the genome is not put in a broader biological context. Why is this genome special? Is it special? How does it compare to other species? Any specific functions? etc etc. The annotation of the genes is mentioned, but nothing is elaborated on it or examined.

Level of Interest

Please indicate how interesting you found the manuscript: Choose an item.

Quality of Written English

Please indicate the quality of language in the manuscript: Choose an item.

Declaration of Competing Interests

Please complete a declaration of competing interests, considering the following questions:

- Have you in the past five years received reimbursements, fees, funding, or salary from an organisation that may in any way gain or lose financially from the publication of this manuscript, either now or in the future?
- Do you hold any stocks or shares in an organisation that may in any way gain or lose financially from the publication of this manuscript, either now or in the future?
- Do you hold or are you currently applying for any patents relating to the content of the manuscript?
- Have you received reimbursements, fees, funding, or salary from an organization that holds or has applied for patents relating to the content of the manuscript?
- Do you have any other financial competing interests?
- Do you have any non-financial competing interests in relation to this paper?

If you can answer no to all of the above, write 'I declare that I have no competing interests' below. If your reply is yes to any, please give details below.

I declare that I have no competing interests

I agree to the open peer review policy of the journal. I understand that my name will be included on my report to the authors and, if the manuscript is accepted for publication, my named report including any attachments I upload will be posted on the website along with the authors' responses. I agree for my report to be made available under an Open Access Creative Commons CC-BY license

(http://creativecommons.org/licenses/by/4.0/). I understand that any comments which I do not wish to be included in my named report can be included as confidential comments to the editors, which will not be published.

I agree to the open peer review policy of the journal

To further support our reviewers, we have joined with Publons, where you can gain additional credit to further highlight your hard work (see: https://publons.com/journal/530/gigascience). On publication of this paper, your review will be automatically added to Publons, you can then choose whether or not to claim your Publons credit. I understand this statement.

Yes Choose an item.
